# Sex/gender differences in orbitofrontal cortex reactivity underlying the associations between stress, social relationships, and problematic alcohol use

**DOI:** 10.1017/S0033291725102250

**Published:** 2025-10-29

**Authors:** Andrea Maxwell, Eric Rawls, Anna Zilverstand

**Affiliations:** 1 Department of Psychiatry & Behavioral Sciences, University of Minnesota, Minneapolis, USA; 2 Department of Psychology, University of North Carolina Wilmington, Wilmington, USA

**Keywords:** affective neuroscience, alcohol use disorder, companionship, emotion regulation, friendship, gender differences, orbitofrontal cortex, sex differences, social neuroscience, stress

## Abstract

**Background:**

Accumulating evidence suggests that stress, social relationships, and sex/gender differences in brain function, particularly of the orbitofrontal cortex (OFC), may drive problematic alcohol use. How these factors interact to effect alcohol use, and if they do so differently in men and women, has yet to be explored.

**Methods:**

Using a subsample of the publicly available Human Connectome Project data consisting of young adults with problematic alcohol use (N = 491; 41.75% women, ≥1 symptom of alcohol abuse/dependence), we used a moderated moderation approach to test whether perceived stress and sex/gender moderated the effect of a multidimensional measure of social relationship quality on drinking levels. We subsequently tested whether OFC function moderated these effects.

**Results:**

We found that in women, higher friendship and companionship had a protective effect on drinking levels, particularly for women under high stress. In contrast, in men, higher friendship and companionship were linked to increased drinking levels under stress. Preliminary evidence suggested that this effect in men was driven by a subgroup of men with higher OFC reactivity to negative emotional faces.

**Conclusions:**

Our findings suggest that women benefit from friendship and companionship as a form of stress-relief in the context of problematic drinking, whereas men do not, supporting the need of interventions that facilitate emotionally supportive, pro-recovery social environments particularly in men. Preliminary evidence further suggests a role of emotional dysregulation in men. Overall, our findings support the importance of developing sex/gender and neurobiologically informed interventions that target stress-related alcohol use.

## Introduction

Historically, men consume more alcohol and report more problematic alcohol use relative to women. This gender gap, however, has dramatically narrowed in the past twenty years. Young women now binge drink at the same rate as men (Shuey et al., 2025), and there is growing concern for alcohol-related problems in women, including diagnosed alcohol use disorder (AUD), alcohol-related hospitalizations, liver disease, and death (White, 2020). Existing research suggests that treatments targeting factors that are more prevalent in women, such as eating disorders and parenting, are more efficacious for women (Greenfield & Grella, 2009). One review of 43 addiction treatment programs found that patients in women-only groups had better outcomes than women in gender-mixed groups (Niv & Hser, 2007). Another study found that gender-responsive resources used in women-only groups, not simply the gender-specific environment, drove positive outcomes (Bride, 2001). Notably, although research on gender-responsive programs in men is limited, a study targeting patients’ social networks in AUD treatment found that facilitating recovery-supportive social networks improved outcomes for men, but not women, suggesting a gendered mechanism (Litt, Kadden, & Tennen, 2015). Therefore, implementing empirically informed, gender-responsive treatment may be key to successful AUD treatment in both women and men.

Although addiction is a neurobiologically based condition (Koob & Volkow, 2010), the translation of neuroscience-based approaches to AUD treatment has been minimal (Verdejo-Garcia et al., 2019). One potential challenge is that alcohol use is driven by a dynamic interplay between an individual’s neurobiological, psychological, and social context, all of which may be affected by sex and gender. Yet, AUD treatments informed by the biopsychosocial model accounting for sex/gender are scarce. Dysregulated stress response may be a key component of this biopsychosocial model, as altered stress reactivity is associated with initiation, maintenance, and relapse of disordered alcohol use (Kwako & Koob, 2017). Acute stress exposure initiates physiological arousal via cortisol release and activates neurocircuits involving the amygdala, hippocampus, insula, orbitofrontal cortex (OFC), and ventromedial prefrontal cortex (vmPFC), among other brain regions, which are key components of brain stress and emotional pathways (Sinha, 2022). Although stress plays a key role in all phases of AUD in both men and women, accumulating evidence suggests that women are more likely to engage in alcohol use as a form of negative reinforcement (i.e. drinking to cope) while men are more likely to engage in alcohol use as a form of positive reinforcement (i.e. social enhancement) (Peltier et al., 2019). Indeed, both preclinical and clinical work suggests that females are more likely to relapse in response to stress during alcohol withdrawal relative to males (Becker & Koob, 2016). Our systematic review of sex/gender differences in the neuroimaging addiction literature found that women with Substance Use Disorders demonstrated greater reactivity in the OFC/vmPFC to stressful cues relative to men, while men demonstrated greater reactivity in the OFC/vmPFC to rewarding cues compared to women (Maxwell, Brucar, & Zilverstand, 2023). These findings converge with the stress-related negative and positive reinforcement models of AUD in women and men, respectively.

Furthermore, social relationship quality (SRQ) plays an integral role in alcohol use. A systematic review found that the characteristics of one’s social network have significant implications for one’s alcohol drinking behavior (Knox et al., 2019). Interestingly, the majority of work examining social relationships and alcohol use in adults has focused on the protective component of social support, defined broadly as an individual’s perception that they are cared for, respected, and a part of a mutually beneficial network of people (Taylor, 2011), against the maladaptive consequences of stress, termed the stress-buffering model (Cohen & Wills, 1985). Investigation of the neurobiological mechanisms of the stress-buffering model have demonstrated that social support modulates threat and stress neural networks (Eisenberger, 2013; Hornstein et al., 2024; Hyde, Gorka, Manuck, & Hariri, 2011; Lin et al., 2023), both of which overlap with alcohol-related reward circuitry (Blaine et al., 2019; Blaine & Sinha, 2017), including the OFC/vmPFC. One report investigating the neurobiological mechanisms underlying the stress-buffering model in alcohol misuse found that individuals with low social support demonstrated greater reactivity in the vmPFC and ventral striatum to alcohol and stress cues relative to those with high social support (Fogelman, Hwang, Sinha, & Seo, 2022). Although current research evidences a role of social support as a powerful modulator of both stress and alcohol use and has started to investigate the underlying brain mechanisms, little work has examined sex/gender differences in these relationships.

Importantly, recent work also indicates gender differences in resiliency mediated by social relationships in AUD. In a data-driven causal model, we found that supportive social relationships had a protective effect on AUD symptom severity by buffering increased negative emotionality in women but not men, converging with previous evidence that social support has a stronger protective effect on alcohol use in adolescent girls than boys (Maxwell, Harrison, Rawls, & Zilverstand, 2022). These data suggest that high quality social relationships are an important gender-specific resilience factor in women with alcohol misuse. A challenge in investigating this further is the complex nature of these social interactions. Current literature often uses the term ‘social support’ so broadly that treatment targets for this multidimensional construct are unclear (Barrera, 1986; Hostinar, Sullivan, & Gunnar, 2014). To address this, the National Institutes of Health (NIH) Toolbox’s SRQ scale conceptualizes relationship quality along the dimensions of social support, companionship, and perceived distress (Cyranowski et al., 2013). Each of these dimensions has been separately associated with alcohol misuse (Gutkind, Gorfinkel, & Hasin, 2022; Li et al., 2021; Pabst, Billaux, Gautier, & Maurage, 2023; Pabst et al., 2020), with substantial research implicating dysregulated prefrontal recruitment, including the OFC/vmPFC, in this relationship (Chester & DeWall, 2014; Le et al., 2021; G. Li et al., 2021; Ohtsubo et al., 2020; Stoddard et al., 2016; Wagels & Hernandez-Pena, 2024). No work that we are aware of, however, has parsed the unique effect of these dimensions on alcohol use or explored sex/gender differences in these associations.

In sum, despite evidence purporting the role of stress, social relationships, and brain function on alcohol misuse, no work that we are aware of has examined sex/gender differences in these factors and their interactions. Using data from the Human Connectome Project (HCP), a publicly available sample of 1,206 young adults, we aim to (1) test whether the effect of SRQ on alcohol drinking levels is moderated by stress and sex/gender, (2) if so, identify which specific dimension of SRQ drives this effect, and (3) test whether this effect is moderated by OFC reactivity to negative stimuli differentially in men and women. We hypothesized that there will be sex/gender differences in the relationship between SRQ, stress, OFC reactivity, and alcohol use. We did not have an a priori hypothesis regarding which SRQ dimension would drive this effect.

## Methods

### Participants

We analyzed the deidentified data from the 1200 Subjects Release (S1200) release of the WU-Minn HCP (N = 1,206, aged 22–35, 54% female), which is publicly available data collected between 2012 and 2015 in Missouri composed of a rich set of self-report, diagnostic, and behavioral measures of emotion, cognition, social function, psychiatric dysfunction, and personality in addition to neuroimaging data. The present analytic sample consisted of participants who endorsed at least one lifetime symptom of DSM-IV-TR abuse or dependence (n = 491, 41.75% women; Supplementary Table 1). Roughly half of the participants endorsed subclinical AUD over the course of his/her lifetime (n = 229; 46.63%), while the other half met criteria for lifetime AUD (n = 262; 53.36%). Within individuals with AUD, 72.52% (38.70% of the entire sample) had mild and 27.48% (14.66% of the sample) had moderate to severe AUD based on their lifetime symptom count (calculated by summing DSM-IV symptoms for alcohol abuse and dependence). A significantly greater proportion of women had subclinical symptoms, while men more frequently had moderate/severe AUD (4–5+ symptoms) (Supplementary Table 1). The HCP consortium does not articulate if and how biological sex or gender was defined; we therefore use the term sex/gender to evaluate differences between binary, self-reported men and women. All study procedures and informed consent forms, including consent to share deidentified data, were approved by the Washington University Institutional Review Board in accordance with the Declaration of Helsinki.

### Measures

#### Perceived stress

The HCP assessed stress using the NIH Perceived Stress Scale from the NIH Toolbox Emotion (Cohen & Janicki-Deverts, 2012; Cohen, Kamarck, & Mermelstein, 1983). Previously published Cronbach’s alpha for this scale range from 0.78 to 0.91 (Lee, 2012).

#### Social relationship quality

The HCP used the self-report NIH SRQ scale from the NIH Toolbox Emotion to assess social relationships (Cyranowski et al., 2013; Salsman et al., 2013). This scale is composed of three subdomains, each with two subscales: companionship (subscales: friendship, loneliness), perceived distress (subscales: perceived hostility and rejection), and social support (subscales: emotional and instrumental support) (see Supplementary Methods and Supplementary Figure 1 for details). The Cronbach’s alpha of these subscales ranges from 0.932 to 0.969 (Cyranowski et al., 2013). We averaged across the six subscales to compute a metric of ‘global’ SRQ.

#### Alcohol abuse and dependence symptom severity and patterns of drinking

Symptoms of alcohol abuse and dependence were assessed using the Semi-Structured Assessment for the Genetics of Alcoholism (Bucholz et al., 1994). Total symptom counts were provided for DSM-IV-TR alcohol abuse and dependence criteria, which was used to identify the analytic sample of problematic drinkers (defined as individuals who endorsed at least one symptom of abuse or dependence). Counts of individual symptoms were not reported by HCP. Drinks consumed per drinking day in the past 12 months (0, 1, 2, 3, 4, 5–6 = 5, 7 + =6) was the outcome in all analyses (Supplementary Figure 2).

### Neuroimaging

#### Angry/fearful faces task

The HCP-emotional processing task was designed to assess brain reactivity to negatively valenced emotional faces (Hariri et al., 2002). In this task, participants matched angry or fearful faces, alternating with blocks during which they matched emotionally neutral shapes (Barch et al., 2013). The contrast between brain reactivity to angry/fearful faces versus emotionally neutral shapes was used in the present analyses and conceptualized as a stress-related response following previous research (Maxwell et al., 2023).

#### Task fMRI acquisition and preprocessing

The HCP consortium collected high-resolution structural and functional 3T MRI data that underwent motion and noise correction, among other preprocessing steps (see Glasser et al., 2013; Uğurbil et al., 2013 for details). Fully analyzed individual (within-subject) task fMRI data were made available as Coefficient of Parameter Estimate (COPE) maps. For the present analyses, we extracted parameter estimates from the angry/fearful faces versus shape contrast COPE maps for each region of interest.

#### Brain region analyses

The OFC and posterior OFC were derived from the bilateral ‘orbital frontal complex’ and ‘posterior OFC complex’ parcels, respectively, from the Glasser Atlas (Glasser et al., 2016). The characteristics of the sample in the neuroimaging analyses (N = 180 women and N = 244 men) did not differ significantly from the behavioral analysis sample (Supplementary Table 2).

### Data analytic plan

**Figure 1.**
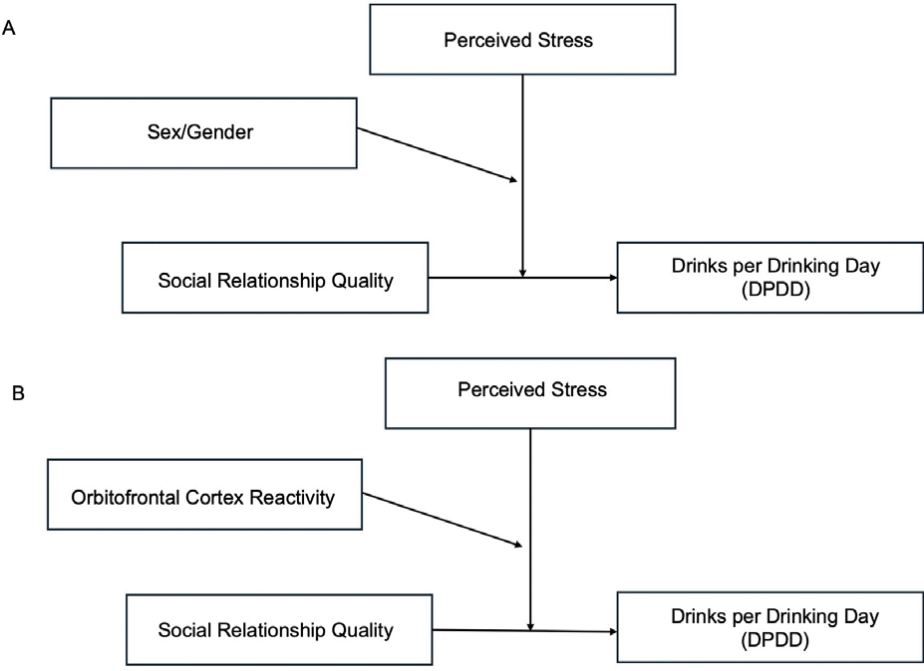
**Analytic schema**. Moderated moderation models testing the effect of social relationship quality on drinks per drinking day, with perceived stress and either (A) sex/gender or (B) orbitofrontal cortex reactivity as the additional moderators.

We used Hayes’ PROCESS (Hayes, 2017) macro version 4.3 in R (version 4.4.1) to test all moderated moderation models (i.e. three-way interactions) (Hayes’ Model 3). We adjusted for multiple comparisons in a hierarchical fashion by applying family wise error correction separately to the primary, secondary, and tertiary ‘families’ of outcomes (see Supplementary Methods for more detail) (Cao & Zhang, 2014; Holm, 1979). Our primary outcome was the effect on drinking by the interaction between ‘global’ SRQ, sex/gender, and stress. Therefore, we first tested a model using ‘global’ SRQ as the focal predictor, sex/gender and stress as moderators, and Drinks Per Drinking Day (DPDD) as the outcome measure (Figure 1A). Given a significant primary outcome, we then tested for secondary effects by subdomain (companionship, perceived distress and social support, p-corrected family wise p < 0.017) as the focal predictor, sex/gender, and stress on drinking levels (DPDD), covarying for the other two subdomains. Third, for significant subdomain effects only, we tested tertiary effects by subscale (friendship, loneliness, p-corrected family wise p < 0.025) as the focal predictor, sex/gender and stress as moderators, with DPDD as the outcome, covarying for all other five subscales (Supplementary Figure 3). Finally, we tested a model separately in men and women using OFC reactivity to the angry/fearful faces versus shapes as a moderator and DPDD as the outcome (Figure 1B, Supplementary Figure 3). Age in years, race (white/non-white), ethnicity (Hispanic/Not Hispanic), and income (binned as in Supplementary Table 1) were included as covariates in all models. When assessing OFC reactivity to stressful socioemotional cues, we additionally controlled for reactivity of bilateral posterior OFC (to angry/fearful faces vs. shapes), given research suggesting that the posterior OFC processes lower-order rather than higher-order (e.g. social) reward (Izuma, Saito, & Sadato, 2008; Sescousse, Redouté, & Dreher, 2010). We applied a robust standard error to correct for heteroscedasticity (HC3), and evaluated stability by generating 5,000 bootstrapped samples to determine a 95% confidence interval around *b* (Hayes & Cai, 2007). All measures were mean-centered prior to statistical analysis, and interactions were conditioned at low (−1 SD), average (mean), and high (+1 SD) levels. All interactions were plotted in R (version 4.4.1) using the *sjPlot* package. The assumptions of linear regression (i.e. outliers, normality, linearity, multicollinearity, and heteroscedasticity) were met (Supplementary Table 3). Sensitivity analyses tested (1) the effect of the insula and dorsal anterior cingulate cortex as moderators on DPDD, given these regions’ role in processing salient stimuli (e.g. stressful triggers, alcohol cues) (Peters, Dunlop, & Downar, 2016) and (2) the effect of alcohol use severity (subthreshold vs. AUD criteria met) on DPDD (Supplementary Methods).

## Results

The primary moderated moderation model testing the effect of global SRQ on DPDD with perceived stress and sex/gender as moderators was significant (F(11,479) = 7.20, p < 0.0001, R^2^ = 0.14), as was the interaction between global SRQ, perceived stress, and sex/gender (*b* = −0.241, t(479) = −2.369, p = 0.018; CI: −0.441, −0.041), which persisted after bootstrapping (BootCI: −0.444, −0.042) (Table 1, Supplementary Figure 4). Conditional effects indicated that, in men with average or higher perceived stress, higher global SRQ was associated with more DPDD (Table 2). Among the three SRQ subdomain models tested (p-corrected: <0.017), only the model testing the effect of companionship on DPDD was significant (F(13,477) = 8.69, p < 0.001, R^2^ = 0.18) with a significant Companionship × Stress × Gender interaction (*b* = −0.207, t(477) = −2.615, p = 0.009; CI: −0.363, −0.052). This effect persisted after bootstrapping (BootCI: −0.376, −0.056) (Table 1, Supplementary Figure 5). Conditional effects for women indicated that while higher companionship was associated with elevated drinking levels in women, this effect was less pronounced in women with high stress, supporting a (small) buffering effect of companionship on stress-related alcohol use in women (Table 2). In contrast, in men, the positive association between companionship and drinking levels was more pronounced in men with high stress levels as compared to men with average or low stress, suggesting that companionship compounded (rather than buffered) the effect of stress on alcohol use in men (Table 2).

**Table 1.** Moderated moderation model parameters

	b	SE (HC3)	t value	p	*b* 95% CI		*b* Bootstrapped 95% CI	
					LL	UL	BootLL	BootUL
**Moderation of the effect of global social relationship quality (SRQ) on drinks per drinking day by perceived stress depending on sex/gender**								
Constant	1.461	0.416	3.509	<0.001	0.643	2.279	0.649	2.255
Global SRQ	0.492	0.226	2.174	0.030	0.047	0.937	0.029	0.910
Perceived stress	0.210	0.178	1.181	0.238	−0.139	0.559	−0.129	0.553
Global SRQ × Perceived stress	0.425	0.154	2.760	0.006	0.123	0.728	0.116	0.727
Sex/gender	−0.761	0.096	−7.906	<0.001	−0.950	−0.572	−0.948	−0.577
Global SRQ × Sex/gender	−0.190	0.153	−1.241	0.215	−0.491	0.111	−0.473	0.124
Perceived stress × Sex/gender	−0.057	0.111	−0.511	0.610	−0.276	0.162	−0.277	0.153
Global SRQ × Perceived stress × Sex/gender	−0.241	0.102	−2.369	0.018	−0.441	−0.041	−0.444	−0.042
Income	0.001	0.023	0.060	0.952	−0.044	0.047	−0.042	0.048
Race	0.067	0.117	0.576	0.565	−0.162	0.296	−0.155	0.288
Ethnicity	−0.173	0.129	−1.340	0.181	−0.427	0.081	−0.423	0.074
Age	−0.013	0.014	−0.960	0.338	−0.041	0.014	−0.040	0.013
**Moderation of the effect of companionship on drinks per drinking day by perceived stress depending on sex/gender**								
Constant	1.318	0.417	3.161	0.002	0.499	2.137	0.492	2.100
Companionship	0.535	0.172	3.108	0.002	0.197	0.873	0.195	0.870
Perceived stress	0.207	0.170	1.220	0.223	−0.127	0.541	−0.112	0.537
Companionship × Perceived stress	0.384	0.126	3.047	0.002	0.136	0.631	0.139	0.640
Sex/gender	−0.720	0.094	−7.670	<0.001	−0.905	−0.536	−0.901	−0.538
Companionship × Sex/gender	−0.103	0.112	−0.926	0.355	−0.323	0.116	−0.318	0.117
Perceived stress × Sex/gender	−0.039	0.104	−0.371	0.711	−0.243	0.166	−0.238	0.154
Global SRQ × Perceived stress × Sex/gender	−0.207	0.079	−2.615	0.009	−0.363	−0.052	−0.376	−0.056
Income	0.006	0.023	0.242	0.809	−0.039	0.050	−0.037	0.052
Race	0.076	0.110	0.684	0.494	−0.141	0.292	−0.128	0.290
Ethnicity	−0.165	0.129	−1.284	0.200	−0.418	0.088	−0.412	0.082
Age	−0.011	0.014	−0.821	0.412	−0.038	0.016	−0.038	0.016
Social support	−0.137	0.066	−2.075	0.039	−0.267	−0.007	−0.261	−0.011
Perceived distress	−0.042	0.064	−0.649	0.517	−0.168	0.084	−0.164	0.085
**Moderation of the effect of friendship on drinks per drinking day by perceived stress depending on sex/gender**								
Constant	1.141	0.425	2.684	0.008	0.306	1.976	0.297	1.926
Friendship	0.458	0.157	2.914	0.004	0.149	0.767	0.169	0.755
Perceived stress	0.135	0.163	0.829	0.408	−0.185	0.455	−0.167	0.456
Friendship × Perceived stress	0.308	0.128	2.402	0.017	0.056	0.561	0.071	0.552
Sex/gender	−0.699	0.092	−7.599	<0.001	−0.879	−0.518	−0.871	−0.519
Friendship × Sex/gender	−0.116	0.093	−1.246	0.213	−0.300	0.067	−0.296	0.062
Perceived stress × Sex/gender	−0.009	0.098	−0.095	0.925	−0.202	0.183	−0.201	0.174
Friendship × Perceived stress × Sex/gender	−0.180	0.078	−2.327	0.020	−0.333	−0.028	−0.335	−0.040
Income	0.012	0.023	0.518	0.605	−0.033	0.056	−0.030	0.057
Race	0.072	0.111	0.646	0.519	−0.146	0.290	−0.129	0.285
Ethnicity	−0.175	0.131	−1.335	0.183	−0.432	0.083	−0.429	0.076
Age	−0.008	0.014	−0.578	0.564	−0.035	0.019	−0.035	0.019
Instrumental support	−0.067	0.054	−1.229	0.220	−0.173	0.040	−0.171	0.035
Emotional support	−0.066	0.060	−1.092	0.276	−0.183	0.052	−0.183	0.043
Loneliness	0.105	0.073	1.435	0.152	−0.039	0.248	−0.028	0.244
Perceived hostility	0.041	0.056	0.719	0.472	−0.070	0.152	−0.070	0.147
Perceived rejection	−0.082	0.071	−1.157	0.248	−0.221	0.057	−0.216	0.057
**Moderation of the effect of loneliness on drinks per drinking day by perceived stress depending on sex/gender**								
Constant	1.286	0.415	3.103	0.002	0.472	2.100	0.477	2.070
Loneliness	0.166	0.166	0.999	0.318	−0.161	0.493	−0.158	0.485
Perceived stress	0.127	0.178	0.716	0.474	−0.222	0.477	−0.199	0.470
Loneliness × Perceived stress	0.325	0.114	2.860	0.004	0.102	0.549	0.099	0.562
Sex/gender	−0.737	0.097	−7.620	<0.001	−0.927	−0.547	−0.924	−0.552
Loneliness × Sex/gender	−0.056	0.105	−0.529	0.597	−0.261	0.150	−0.253	0.152
Perceived stress × Sex/gender	0.000	0.110	−0.001	1.000	−0.215	0.215	−0.215	0.202
Loneliness × Perceived stress × Sex/gender	−0.169	0.072	−2.361	0.019	−0.310	−0.028	−0.320	−0.027
Income	0.006	0.023	0.265	0.791	−0.039	0.051	−0.036	0.052
Race	0.073	0.110	0.663	0.508	−0.144	0.290	−0.127	0.289
Ethnicity	−0.167	0.128	−1.303	0.193	−0.419	0.085	−0.420	0.082
Age	−0.009	0.014	−0.659	0.511	−0.036	0.018	−0.036	0.017
Instrumental support	−0.071	0.053	−1.333	0.183	−0.175	0.034	−0.173	0.031
Emotional support	−0.048	0.058	−0.818	0.414	−0.162	0.067	−0.163	0.060
Friendship	0.286	0.057	5.050	<0.001	0.174	0.397	0.179	0.391
Perceived hostility	0.024	0.055	0.435	0.664	−0.085	0.133	−0.084	0.132
Perceived rejection	−0.075	0.069	−1.084	0.279	−0.210	0.061	−0.208	0.061
**Moderation of the effect of loneliness on drinks per drinking day by perceived stress depending on orbitofrontal cortex reactivity in men**								
Constant	0.495	0.652	0.760	0.448	−0.789	1.779	−0.847	1.648
Loneliness	0.047	0.100	0.469	0.640	−0.149	0.243	−0.145	0.243
Perceived stress	0.094	0.094	1.007	0.315	−0.090	0.279	−0.081	0.273
Loneliness × Perceived stress	0.151	0.059	2.545	0.012	0.034	0.267	0.044	0.271
OFC reactivity	−0.279	0.134	−2.083	0.038	−0.543	−0.015	−0.525	−0.013
Loneliness × OFC reactivity	−0.026	0.086	−0.302	0.763	−0.195	0.143	−0.189	0.145
Perceived stress × OFC reactivity	0.061	0.091	0.670	0.503	−0.118	0.240	−0.110	0.244
Loneliness × Perceived stress × OFC reactivity	0.120	0.060	2.019	0.045	0.003	0.237	−0.004	0.243
Income	0.010	0.032	0.321	0.748	−0.053	0.074	−0.050	0.072
Race	0.377	0.187	2.016	0.045	0.009	0.746	0.043	0.758
Ethnicity	−0.126	0.198	−0.637	0.525	−0.517	0.264	−0.492	0.274
Age	−0.027	0.022	−1.201	0.231	−0.070	0.017	−0.066	0.017
Instrumental support	−0.101	0.083	−1.221	0.223	−0.265	0.062	−0.263	0.049
Emotional support	−0.010	0.092	−0.102	0.919	−0.192	0.173	−0.180	0.172
Friendship	0.291	0.081	3.594	<0.001	0.131	0.450	0.140	0.446
Perceived hostility	0.055	0.078	0.702	0.483	−0.099	0.209	−0.102	0.209
Perceived rejection	−0.073	0.105	−0.697	0.487	−0.279	0.133	−0.260	0.133
Posterior OFC reactivity	0.201	0.120	1.677	0.095	−0.035	0.436	−0.041	0.418

Abbreviations: CI, confidence interval; SE(HC3), heteroscedasticity-robust standard error 3; Boot, bootstrapped; LL. lower limit; UL, upper limit.

**Table 2.** Conditional effects of moderated moderation models

		b	SE (HC3)	t value	p	*b* 95% CI	
						LL	UL
Conditional effects of global social relationship quality at levels of perceived stress and sex/gender							
**Perceived stress**	**Sex/gender**						
Low	Men	0.117	0.119	0.989	0.323	−0.116	0.351
Low	Women	0.168	0.171	0.983	0.326	−0.168	0.505
Average	Men	0.302	0.096	3.131	0.002	0.113	0.492
Average	Women	0.112	0.119	0.939	0.348	−0.123	0.347
High	Men	0.487	0.116	4.201	<0.001	0.259	0.714
High	Women	0.056	0.104	0.536	0.592	−0.149	0.260
Conditional effects of companionship at levels of perceived stress and sex/gender							
**Perceived stress**	**Sex/gender**						
Low	Men	0.255	0.102	2.497	0.013	0.054	0.456
Low	Women	0.359	0.127	2.833	0.005	0.110	0.608
Average	Men	0.432	0.083	5.227	<0.001	0.269	0.594
Average	Women	0.328	0.095	3.469	0.001	0.142	0.514
High	Men	0.608	0.098	6.202	<0.001	0.415	0.801
High	Women	0.297	0.090	3.305	0.001	0.121	0.474
Conditional effects of friendship at levels of perceived stress and sex/gender							
**Perceived stress**	**Sex/gender**						
Low	Men	0.214	0.095	2.260	0.024	0.028	0.399
Low	Women	0.278	0.099	2.801	0.005	0.083	0.473
Average	Men	0.342	0.079	4.324	<0.001	0.186	0.497
Average	Women	0.225	0.072	3.122	0.002	0.084	0.367
High	Men	0.470	0.103	4.573	<0.001	0.268	0.671
High	Women	0.173	0.075	2.305	0.022	0.026	0.321
Conditional effects of loneliness at levels of perceived stress and sex/gender							
**Perceived stress**	**Sex/gender**						
Low	Men	−0.046	0.100	−0.455	0.649	−0.242	0.151
Low	Women	0.068	0.112	0.605	0.546	−0.153	0.289
Average	Men	0.111	0.084	1.325	0.186	−0.053	0.275
Average	Women	0.055	0.091	0.608	0.544	−0.123	0.234
High	Men	0.267	0.096	2.771	0.006	0.078	0.456
High	Women	0.042	0.096	0.444	0.657	−0.145	0.230
Conditional effects of loneliness at levels of perceived stress and orbitofrontal cortex reactivity in men							
**Perceived stress**	**OFC reactivity**						
Low	Low	0.042	0.159	0.265	0.792	−0.271	0.355
Low	Average	−0.104	0.118	−0.884	0.378	−0.336	0.128
Low	High	−0.250	0.152	−1.647	0.101	−0.549	0.049
Average	Low	0.073	0.147	0.492	0.623	−0.218	0.363
Average	Average	0.047	0.100	0.469	0.640	−0.149	0.243
Average	High	0.021	0.113	0.183	0.855	−0.202	0.243
High	Low	0.103	0.173	0.595	0.553	−0.238	0.445
High	Average	0.197	0.114	1.734	0.084	−0.027	0.422
High	High	0.292	0.137	2.121	0.035	0.021	0.562

Abbreviations: CI, confidence interval; SE(HC3), heteroscedasticity-robust standard error 3; LL, lower limit; UL, upper limit.

**Figure 2.**
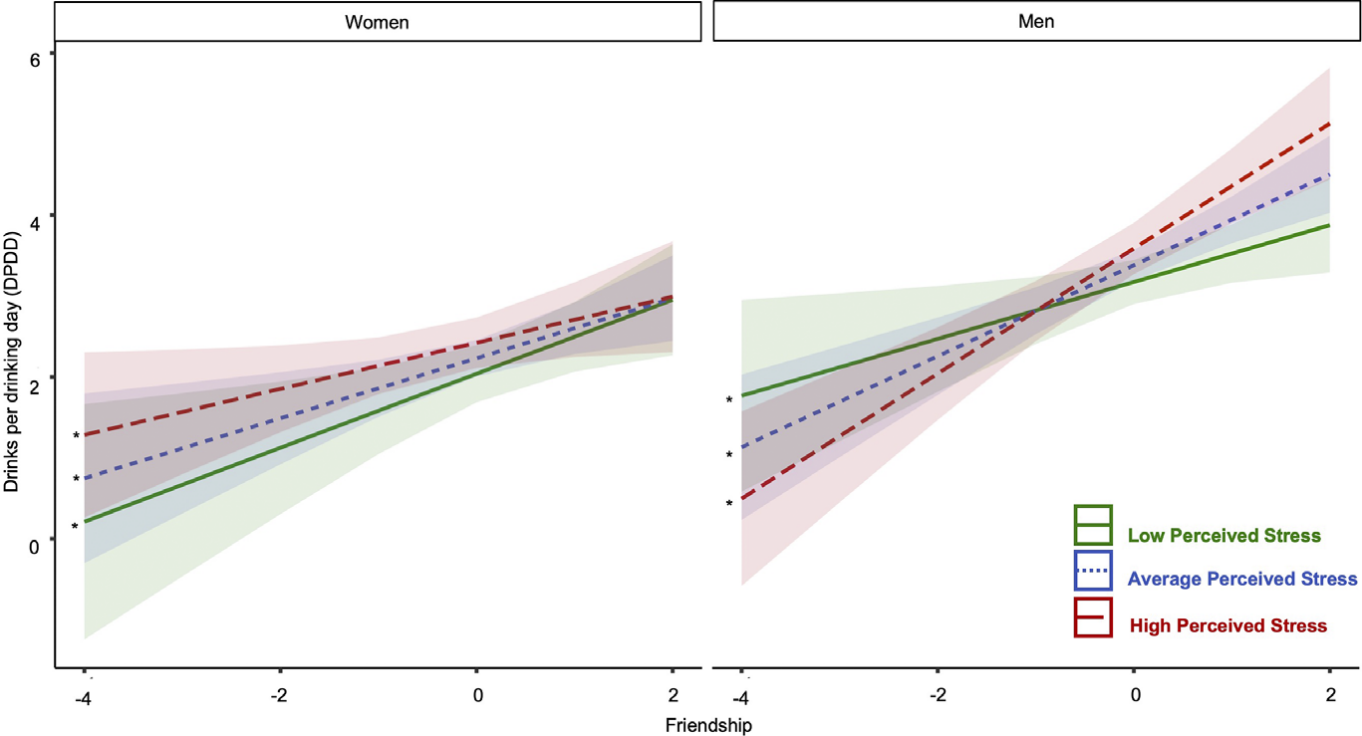
**Predicted values of drinks per drinking day by friendship, perceived stress, and sex/gender**. Predictor and outcome variables were scaled for analysis; raw outcome values are shown here for interpretability; * = statistically significant (p < 0.05) conditional effect.

Given the significant companionship interaction effect, we then tested for effects of its subscales friendship and loneliness (p-corrected: p < 0.025). The friendship model was significant (F(16,474) = 6.85, p < 0.0001, R^2^ = 0.19) with a significant Friendship × Stress × Gender interaction (*b* = −0.180, t(474) = −2.327, p = 0.020; CI: −0.333, −0.028) that survived bootstrapping (Boot CI: −0.335, −0.040) (Table 1, Figure 2). Conditional effects indicated a protective effect of friendship on drinking levels in women with high stress, whereas high friendship compounded the effect of high stress on drinking levels in men (Table 2, Figure 2). The loneliness model was also significant (F(16,474) = 7.90, p < 0.0001, R^2^ = 0.19), with a significant Loneliness × Stress × Gender interaction (*b* = −0.169, t(474) = −2.361, p = 0.019; CI: −0.310, −0.028) that survived Bonferroni correction (significant at p < 0.05/two tests; p < 0.025) and bootstrapping (BootCI: −0.320, −0.027) (Table 1, Supplementary Figure 6). This result was driven by a significant effect in men with high stress, again indicating that low levels of loneliness compounded the effect of high stress on drinking levels specifically in men (Table 2, Supplementary Figure 6).

**Figure 3.**
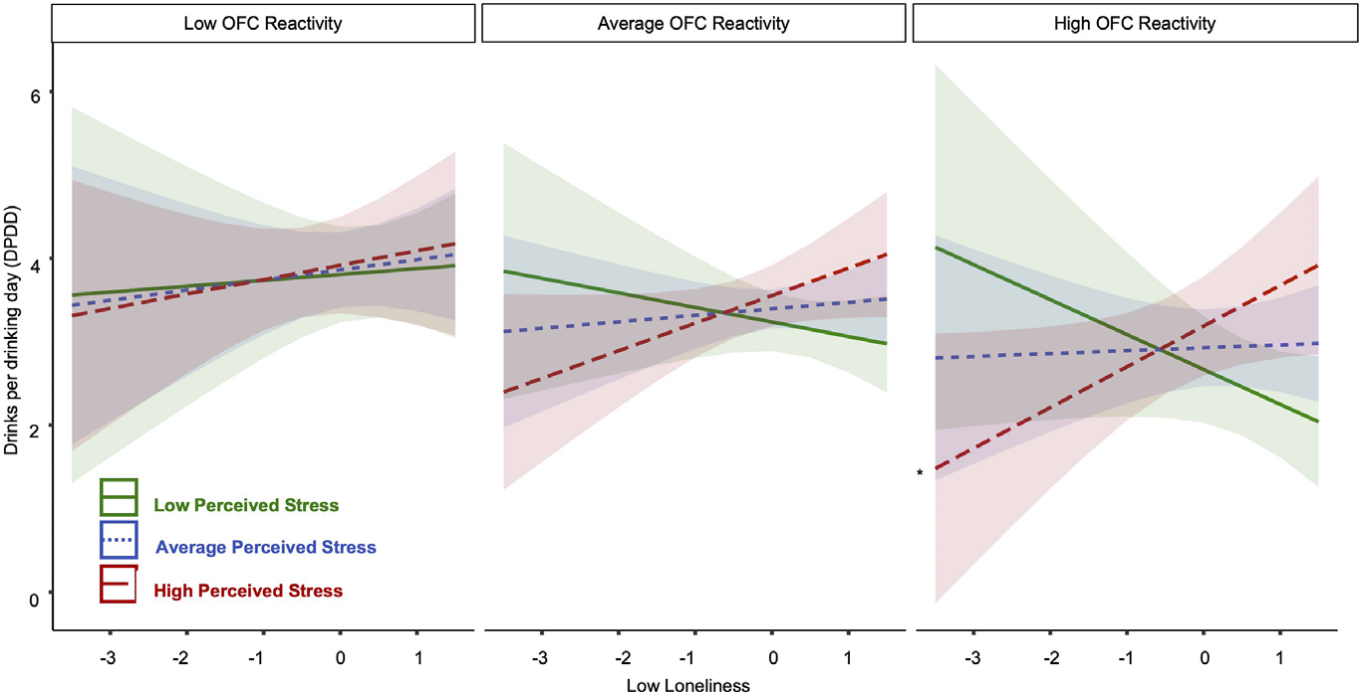
**Predicted values of drinks per drinking day by loneliness, perceived stress, and orbitofrontal cortex reactivity to emotional faces in men**. Predictor and outcome variables were scaled for analysis; raw outcome values are shown here for interpretability. OFC = orbitofrontal cortex; * = statistically significant (p < 0.05) conditional effect.

We then tested in men and women separately whether OFC reactivity to angry/fearful faces (vs. shapes) and perceived stress affected the relationship between either loneliness or friendship on DPDD. We found that only the model with loneliness as the focal predictor in men was significant (F(17,226) = 3.48, p < 0.0001, R^2^ = 0.17). There were no significant effects for OFC reactivity in women. In men, we found a main effect of OFC reactivity to angry/fearful faces versus shapes (*b* = −0.279, t(226) = −2.083, p = 0.038; CI: −0.543, −0.015) that survived bootstrapping (BootCI: −0.525, −0.013). The Loneliness × OFC Reactivity × Perceived Stress interaction in men was also significant (*b* = 0.120, t(226) = 2.019, p = 0.045; CIs: 0.003, 0.237), although this effect did not persist after bootstrapping (BootCI: −0.004, 0.243) (Table 1, Figure 3, Supplementary Table 4). Conditional effects indicated a compounding effect of low loneliness on DPDD specifically in men with high stress and high OFC reactivity to angry/fearful faces (Table 2, Figure 3, Supplementary Table 4). The additional sensitivity analysis conducted for the insula and dorsal anterior cingulate revealed no significant effects (Supplementary Methods).

Finally, as expected, the subthreshold group reported significantly lower alcohol use levels compared to the AUD group (Supplementary Table 5, Supplementary Figures 7–8). Sensitivity analyses of each three-way interaction tested separately in the subthreshold and AUD group demonstrated that the loneliness effect reached significance only in the AUD group (*b* = −0. 216, t(245) = −2.345, p = 0.0197). Both the companionship (*b* = −0.191, t(248) = −1.906, p = 0.058) and OFC effects in men (*b* = 0.146 t(119) = 1.774, p = 0.079) trended toward significance in the AUD group only. Overall, as expected, effects were thus stronger in the AUD compared to the subthreshold group, though the observed patterns were strikingly similar between groups (Supplementary Figures 9–13). There were also no significant interactions in the tested interaction models when accounting for group (subthreshold vs. AUD) (Supplementary Figures 14–18).

## Discussion

Here, we used a biopsychosocial model to examine sex/gender differences in the effect of perceived stress, SRQ, and brain function on problematic alcohol use. We tested a series of moderated moderation models to identify (1) whether there are sex/gender differences in the effects of SRQ and stress on alcohol use, (2) which dimensions of SRQ drives these effects, and (3) whether these effects are moderated by OFC reactivity to negative socioemotional stimuli differently in men and women. We hypothesized that there are sex/gender differences in the complex ways these factors interact to affect drinking. In support of this hypothesis, we found that in women only, higher companionship and friendship levels were protective against the effect of high stress levels on drinking. In contrast, in men, higher companionship and friendship and lower loneliness promoted drinking particularly with high stress levels, thus compounding the effect of stress on drinking levels. This effect was particularly salient in a subgroup of men with high OFC reactivity to negative emotional faces.

In summary, we demonstrated striking sex/gender differences in the psychosocial factors underlying problematic drinking. While companionship and friendship buffered the effects of stress on drinking in women, these were actually risk factors in men, particularly at high stress levels. Overall, our findings align with the existing model of gender differences in stress-related drinking behavior, which suggests that women tend to consume alcohol as a form of negative reinforcement (i.e. drinking to cope with stressors) (Peltier et al., 2019). We demonstrated that women with higher stress levels drank more than those with lower stress levels. However, we also extend this model by integrating social factors and demonstrating a buffering effect of companionship and friendship on stress-related alcohol use specifically to women. This buffering effect in women is generally in line with previous (non-gender-specific) work demonstrating the importance of social support as a buffer against upregulated stress and threat reactivity (Cohen & Wills, 1985; Eisenberger et al., 2007; Fogelman et al., 2022; Hyde et al., 2011), including our previous research (Maxwell et al., 2022).

In men, we found that in contrast to women, companionship and friendship were linked to higher levels of drinking, driven by a subgroup of men who was highly OFC reactive to negative socioemotional stimuli. This subgroup of men was therefore similar to women with regard to their OFC reactivity to stressful stimuli, but did not profit from social support as a buffering factor. In men, the promotional effect of friendship/companionship on drinking thus seemed to outweigh the buffering effect of social support, such that men did not benefit from friendship/companionship as a mechanism to relieve stress, but rather seemed to engage in socially driven alcohol use as stress relief. This socially driven alcohol use is consistent with the previously proposed sex differences model suggesting that men engage in alcohol use as a form of positive reinforcement (Peltier et al., 2019). Prior research also found that men report less intimacy and emotional support in friendships (21% of men as compared to 41% of women reported receiving emotional support from friends in the past week) (Cox, 2021). Indeed, despite changing gender norms related to alcohol use (Abbott-Chapman, Denholm, & Wyld, 2008; Slade et al., 2016), traditionally gendered roles modulating social behavior in friendships have not changed over the past decades (Gil, 2023; Liebler & Sandefur, 2002). Women have been and remain the primary provider of emotional support in relationships, which is especially important since both men and women tend to primarily engage in same-sex friendships (Baumgarte & Nelson, 2009; Gillespie, Frederick, Harari, & Grov, 2015). Accordingly, prior studies report that men endorse higher levels of alcohol drinking in a social context primarily when drinking with other men (Mehta, Alfonso, Delaney, & Ayotte, 2014; Thrul, Labhart, & Kuntsche, 2017). Additionally, while marriage has a protective effect on alcohol use particularly in men, marriage rates have also been declining (Salvatore, Gardner, & Kendler, 2020). In summary, gendered social roles seem to contribute to striking gender differences in the promotive (vs. protective) effect of friendship on alcohol use in men (vs. women).

Finally, our current work explored neurobiological mechanisms underlying sex/gender differences in stress-related drinking. Previous work provides ample evidence that altered OFC function is common in addiction and is linked to emotion dysregulation (Chase, Kumar, Eickhoff, & Dombrovski, 2015; Johnson, Elliott, & Carver, 2020; Schoenbaum, Chang, Lucantonio, & Takahashi, 2016; Sescousse, Caldú, Segura, & Dreher, 2013; Zilverstand, Huang, Alia-Klein, & Goldstein, 2018). Here, we found evidence that stress-related drinking behavior in men was driven by a subgroup of men with increased OFC reactivity to negative emotional faces, suggesting that this subgroup may be particularly vulnerable to alcohol misuse via increased emotion dysregulation in a socioemotional context. In agreement with this finding, men with a history of depression and suicide attempts, compared to those without any suicide attempts, exhibit heightened OFC reactivity to angry versus neutral faces (Jollant et al., 2008). Similarly, a study of social drinkers found that negative urgency mediated the relationship between increased OFC reactivity to negative emotional faces and self-reported risk-taking behavior (Cyders et al., 2015). Contextualized with our findings, these data suggest that a dysregulated, OFC-related stress response to negative socioemotional stimuli may underlie risk for problematic alcohol use in this subgroup of men.

Overall, our findings suggest that sex/gender and neurobiologically informed treatments may be beneficial in AUD. In men, traditional social gender norms encourage alcohol use (Iwamoto et al., 2011; Nolen-Hoeksema, 2004; Zamboanga et al., 2017), and men may therefore often be restricted to gendered drinking environments to maintain friendships and connect with their support system for stress relief (e.g. drinking while watching sports at a friend’s house) (Nordin, Degerstedt, & Granholm Valmari, 2024; Paradis, 2011). This social norm may be a particular obstacle for men with problematic drinking. Kelly and Hoeppner found that Alcoholics Anonymous may be more effective in men relative to women by facilitating connections with pro-recovery friends and increasing self-efficacy in managing high-risk drinking social situations in men (Kelly & Hoeppner, 2013). Additionally, enhancing skills around initiating and maintaining emotionally supportive friendships may be an important intervention for men with AUD. Indeed, only 30% of American men reported having had a private conversation during which they shared personal problems or feelings in the past week, compared to 48% of American women (Cox, 2021). Therefore, supporting socialization that facilitates emotionally supportive companionship outside of alcohol-related environments (i.e. sober activities) may be particularly beneficial in men, especially those who struggle with emotion dysregulation. Furthermore, women may also benefit from gender-specific treatments. We found that higher friendship was protective against elevated drinking in stressful situations in women, which may be primarily driven by friendships with other women. Indeed, one study of an effective women-focused group therapy found that women-focused groups elicited greater ‘affiliative statements’ relative to mixed-gender group drug counseling, suggesting that increased affiliation with a support group is one mechanism through which these treatments work for women (Greenfield et al., 2014; Greenfield et al., 2007; Sugarman et al., 2016). Finally, our findings suggest that regulating OFC/vmPFC reactivity may be a reasonable target to reduce stress-related alcohol use. Indeed, normalizing OFC function may be a key neurobiological mechanism underlying mindfulness-based relapse prevention or neuromodulation in AUD (Bowen et al., 2014; Hanlon et al., 2017; Li et al., 2017; Witkiewitz, Lustyk, & Bowen, 2013; Zeidan, Baumgartner, & Coghill, 2019).

## Limitations

Interpretation of this work should account for limitations. First, the HCP did not account for the gender composition of friend groups in this sample. Second, the available neuroimaging data only included contrasts of brain reactivity to angry/fearful faces (a marker of negative reinforcement) versus shapes, but no equivalent in the positive reinforcement domain. We therefore could not directly test if OFC reactivity to reinforcing stimuli would predict alcohol use in men. Third, likely due to the limited sample size and smaller effect sizes in neuroimaging analyses, the moderating effect of OFC reactivity in men did not survive bootstrapping, while the effect in women was not significant, precluding strong conclusions on this specific analysis. The OFC is particularly susceptible to artifacts due to its spatial proximity to the nasal cavities (Stenger, 2006). HCP, however, did implement several technical advances to improve data quality and mitigate this dropout, including shorter echo times, thinner slice acquisitions, and parallel (multiband) imaging, among others and performed susceptibility artifact correction during preprocessing (Glasser et al., 2013; Uğurbil et al., 2013). Fourth, the HCP included the total number of AUD symptoms endorsed over a participant’s lifetime and whether they have ever met criteria for AUD; however, individual AUD symptom counts were not available for further characterization of the sample.

## Conclusion

This study, for the first time to our knowledge, investigates the complex interactions between sex/gender, stress, social relationships, and brain function, developing a biopsychosocial model of problematic alcohol use. We found sex/gender differences that both reiterate and extend existing models of problematic alcohol use. Specifically, we found that companionship and friendship had a protective effect against stress-related drinking in women but compounded the effect of stress on drinking in men. These findings suggest that developing treatments that facilitate emotionally supportive, pro-recovery social environments may be particularly important in men. We further found preliminary evidence that this effect in men may be driven by a subgroup of men with OFC hyperreactivity to negative social–emotional stimuli.

## Supporting information

Maxwell et al. supplementary material Maxwell et al. supplementary material

## Supplementary material

The supplementary material for this article can be found at http://doi.org/10.1017/S0033291725102250.
