# Supplementary material for: Sex/gender differences in orbitofrontal cortex reactivity underlying the associations between stress, social relationships, and problematic alcohol use
Source: Psychol Med. 2025 Oct 29;55:e326. doi: 10.1017/S0033291725102250 (PMC12834410; doi:10.1017/S0033291725102250)
Supplement: Maxwell et al. supplementary material [file S0033291725102250sup001.pdf]

Title: Sex/gender differences in orbitofrontal cortex reactivity underlying the associations between stress, social relationships, and problematic alcohol use

Authors: Maxwell AM[1], Rawls Eric[2], Zilverstand A[1]

[1] Department for Psychiatry and Behavioral Sciences, University of Minnesota, Minneapolis, Minnesota.

[2] Department of Psychology, University of North Carolina Wilmington

Supplemental Material

Supplemental Methods

Social Relationship Quality

The friendship subscale is an 8-item measure of perceived availability of companions with whom to interact or affiliate. The loneliness subscale is a 5-item measure of subjective loneliness. The perceived hostility and rejection subscales are 8-item measures assessing perceptions of daily social interactions. Emotional support and instrumental support are 8-item measures assessing the perception that people in one's social network are available to empathetically listen to one's problems and provide material/functional aid, respectively. For all subscales, higher scores indicate higher levels of the construct; however, loneliness, perceived hostility, and perceived rejection were reverse-scored in the present analyses, such that higher scores generally indicated higher quality of relationships, to facilitate the comparison across scales. We averaged across the six subscales to compute a metric of 'global' social relationship quality and the sum scores of each subscale within a single subdomain to generate a metric of companionship, social support, and perceived distress.

Correction for Multiple Comparisons

We applied Bonferroni correction to account for multiple comparisons. Bonferroni is a well-established conservative approach to reducing family wise error rate (ie, reducing false positives) in which the Bonferroni-corrected significance level is determined by dividing $\alpha = 0.05$ by the number of tests performed within a "family", which is composed of hypotheses that are intended to be interpreted together (Cao & Zhang, 2014; Staff & Zurakowski 2020). In the present analyses, Bonferroni correction was applied to each "family" of outcomes as detailed below.

i. Family 1 (Primary outcome): Global SRQ X Sex/gender X Stress interaction effect
   a. p=0.05/1 test = p=0.05 threshold
ii. Family 2 (Secondary outcomes, conducted only if Family 1 is significant): Companionship X Sex/gender X Stress + Perceived Distress X Sex/gender X Stress + Social Support X Sex/gender X Stress
   a. p=0.05/3 tests = p=0.017 threshold
iii. Family 3 (Tertiary outcomes, conducted only if a Family 2 test is significant): Friendship X Stress X Sex/gender + Loneliness X Stress X Gender
   a. p=0.05/2 = p=0.025 threshold
iv. Family 4 (Theoretically separate model): Loneliness X OFC Reactivity X Stress in men
   a. p=0.05/1 = p=0.05 threshold

Sensitivity analyses

To generate the bilateral dorsal anterior cingulate cortex (dACC) region-of-interest used in the sensitivity analysis, we averaged across five Glasser parcels from the left and right hemispheres: Anterior 24 prime (A24pr), Area 33 prime (33pr), Area Posterior 24 prime (p24pr), Ventral Area (24dv), and Area p32 prime (p32pr).To generate the bilateral insula region-of-interest, we averaged across two bilateral Glasser Middle Insular Area (MI) and Anterior agranular insula complex (AAIC).

Supplemental Table 1. Behavioral Analyses Sample Characteristics

	Total (N=491)	Men (N=286)	Women (N=205)	Sex/Gender Difference *p*
**Demographics**				
Average Age in Years (SD)	28.74 (3.50)	28.38 (3.52)	29.23 (3.41)	0.007
Race (%)				
American Indian/Alaskan Native	0.20	0.35	0.00	1.000
Asian/Native Hawaiian/ Pacific Islander	4.28	4.55	3.90	0.904
Black or African American	11.81	11.19	12.68	0.716
White	78.41	78.67	78.05	0.957
More than one	2.85	3.15	2.44	0.850
Unknown/Not reported	2.44	2.10	2.93	0.772
Ethnicity (%)				
Hispanic/Latino	11.00	12.94	8.29	0.140
Unknown or Not Reported	0.41	0.70	0.00	0.630
Income (%)				
<$10,000	6.72	6.64	6.83	1.000
$10K-19,999	7.74	9.44	5.37	0.135
$20K-29,999	14.05	14.34	13.66	0.935
$30K-39,999	13.03	11.19	15.61	0.194
$40K-49,999	9.98	9.09	11.22	0.533
$50-74,999	20.37	18.88	22.44	0.394
$75K-99,999	10.59	11.54	9.27	0.511
≥ $100,0000	17.52	18.88	15.61	0.412
**Alcohol Use Measures**				
Average drinks per drinking day (DPDD)	2.87 (1.65)	3.30 (1.69)	2.28 (1.38)	< 0.001
Alcohol Use Disorder (AUD) Severity (%)				
Subclinical (1 symptom)	46.63	41.96	53.17	0.018
Mild AUD (2-3 symptoms)	38.70	38.11	39.51	0.826
Moderate/Severe AUD (4-5+ symptoms)	14.66	19.93	7.32	<0.001
**Social Relationship Quality Subscales Averages (SD)**				
Global SRQ	59.68 (6.30)	59.38 (6.25)	60.11 (6.35)	0.208
Instrumental Support	47.94 (9.25)	48.10 (9.38)	47.72 (9.08)	0.654
Emotional Support	51.21 (9.52)	50.49 (9.65)	52.22 (9.28)	0.046
Friendship	50.47 (8.60)	50.50 (8.41)	50.42 (8.89)	0.919
Loneliness	51.49 (8.85)	51.24 (9.24)	51.86 (8.30)	0.441
Perceived Hostility	49.37 (8.39)	50.15 (8.25)	48.29 (8.47)	0.016
Perceived Rejection	49.17 (8.93)	49.47 (8.99)	48.74 (8.85)	0.373
**Average Perceived Stress (SD)**	**49.08 (9.47)**	**48.20 (9.23)**	**50.30 (9.68)**	**0.016**

Welch’s t-test and 2-sample test for equality of proportions with Yates continuity correction used for statistical comparisons. SD = Standard Deviation

Supplemental Table 2. Neuroimaging Analyses Sample Characteristics

	Men (N=244)	Behavioral vs Neuroimaging Samples: Men *p*	Women (N=180)	Behavioral vs Neuroimaging Samples: Women *p*
**Demographics**				
Average Age in Years (SD)	28.35 (3.54)	0.935	29.12 (3.50)	0.740
Race (%)				
American Indian/Alaskan Native	0.41	1.000	0.00	1.000
Asian/Native Hawaiian/Other Pacific Island	4.10	0.414	3.89	1.000
Black or African American	9.02	0.497	13.33	0.970
White	81.56	0.472	77.22	0.943
More than one	2.87	1.000	2.78	1.000
Unknown/Not reported	2.05	1.000	2.78	1.000
Ethnicity (%)				
Hispanic/Latino	13.11	1.000	8.33	1.000
Unknown or Not Reported	0.82	1.000	0.00	1.000
Income (%)				
<$10,000	6.97	1.000	7.22	1.000
10K-19,999	8.20	0.727	4.44	0.857
20K-29,999	15.16	0.885	13.33	1.000
30K-39,999	10.66	0.955	17.22	0.773
40K-49,999	8.61	0.966	10.00	0.825
50-74,999	18.44	0.986	24.44	0.731
75K-99,999	11.89	1.000	9.44	1.000
≥100,0000	20.08	0.812	13.89	0.741
**Alcohol Use Measures**				
Average drinkers per drinking day	3.25	0.730	2.29 (1.39)	0.939
Alcohol Use Disorder (AUD) Severity (%)				
Subclinical (1 symptom)	43.85	0.725	52.78	1.000
Mild AUD (2-3 symptoms)	34.02	0.375	40.00	1.000
Moderate/Severe AUD (4-5+ symptoms)	22.13	0.608	7.22	1.000
**Social Relationship Quality Subscale Averages (SD)**				
Global Social Relationship Quality	59.44 (6.18)	0.909	60.22 (6.24)	0.852
Instrumental Support	48.15 (9.32)	0.948	47.60 (8.99)	0.900
Emotional Support	50.57 (9.45)	0.925	52.32 (9.28)	0.911
Friendship	50.91 (8.27)	0.582	50.68 (8.75)	0.779
Loneliness	51.25 (9.15)	0.991	51. 68 (8.19)	0.842
Perceived Hostility	50.22 (8.05)	0.917	48.13 (8.31)	0.856
Perceived Rejection	49.31 (8.86)	0.833	48.38 (8.95)	0.692
**Average Perceived Stress Scale**	47.92 (9.09)	0.729	50.31 (9.79)	0.992

Welch's t-test and 2-sample test for equality of proportions with Yates continuity correction used for statistical comparisons. SD = Standard Deviation.

Supplemental Table 3. Correlations between social relationship quality indices

	1	2	3	4	5	6	7	8	9	10	11
1. Friendship	1.00										
2. Loneliness	-0.56	1.00									
3. Instrumental Support	0.34	-0.43	1.00								
4. Emotional Support	0.55	-0.51	0.50	1.00							
5. Perceived Stress	-0.38	0.59	-0.32	-0.42	1.00						
6. Perceived Hostility	-0.28	0.36	-0.15	-0.34	0.41	1.00					
7. Perceived Rejection	-0.47	0.65	-0.35	-0.56	0.54	0.59	1.00				
8. Global Social Relationship Quality	0.70	-0.76	0.61	0.77	-0.59	-0.71	-0.75	1.00			
9. Social Support	0.51	-0.54	0.87	0.87	-0.43	-0.28	-0.53	0.80	1.00		
10. Companionship	0.88	-0.88	0.43	0.60	-0.55	-0.36	-0.63	0.83	0.60	1.00	
11. Perceived Distress	0.42	-0.56	0.28	0.51	-0.53	-0.89	-0.89	0.82	0.45	0.56	1.00

Pearson correlations between all subscales, subdomains, and global social relationship quality included in the behavioral analyses (N=491). Variables included together in a single model are grouped by color.

Supplemental Table 4. Sample characteristics by orbitofrontal cortex reactivity

	Low OFC (N=27)	Average OFC (N=177)	High OFC (N=40)	*p*	Post-hoc test
**Demographics**					
Average Age in Years	29.81 (4.15)	28.26 (3.38)	27.78 (3.60)	0.054	---
Race (%)					
American Indian/Alaskan Native	0.00	0.00	2.50	0.275	---
Asian/Native Hawaiian/Other Pacific Island	3.70	5.08	0.00	0.357	---
Black or African American	11.11	9.04	7.50	0.882	---
White	81.48	80.2	87.50	0.962	---
More than one	3.70	2.82	2.50	0.886	---
Unknown/Not reported	0.00	2.82	0.00	0.480	---
Ethnicity (%)					
Hispanic/Latino	14.81	14.69	5.00	0.246	---
Unknown or Not Reported	0.00	0.56	2.50	0.459	---
Income (%)					
<$10,000	7.41	7.3	5.00	0.924	---
10K-19,999	3.70	8.47	10.00	0.604	---
20K-29,999	18.52	14.12	17.50	0.744	---
30K-39,999	7.41	11.30	10.00	0.901	---
40K-49,999	3.70	10.17	5.00	0.383	---
50-74,999	25.93	18.64	12.50	0.378	---
75K-99,999	18.52	12.43	5.00	0.253	---
≥100,0000	14.81	17.51	37.50	0.034	High OFC > Average OFC: X2(1, N= 217) = 35.57, p<-0.001<br>High OFC > Low OFC: X2(1, N= 204) = 51.695 ; p <0.001
**Alcohol Use Measures**					
Average drinkers per drinking day (DPDD)	4.04 (1.72)	3.17 (1.64)	3.08 (1.70)	0.033	Low OFC > Average OFC: Mean difference: 0.87; 95% CI [0.060, 1.68], p=0.032<br>Low OFC > High OFC: Mean difference: 0.96; 95% CI [-0.01, 1.94]; p=0.054
Alcohol Use Disorder (AUD) Severity (%)					
Subclinical (1 symptom)	33.33	46.33	40.00	0.388	---
Mild AUD (2-3 symptoms)	59.26	41.24	37.50	0.163	---
Moderate/Severe AUD (4-5+ symptoms)	7.41	12.43	22.50	0.155	---
**Social Relationship Quality Subscale Averages (SD)**					
Global Social Relationship Quality	59. 91 (5.82)	58.97 (6.24)	61.17 (5.96)	0.118	---
Instrumental Support	47. 85 (9.72)	47.68 (9.00)	50. 43 (10.32)	0.239	---
Emotional Support	50. 77 (9.01)	50. 05 (9.57)	52. 75 (9.11)	0.264	---
Friendship	51.90 (9.15)	50. 49 (8.20)	52.09 (8.01)	0.434	---
Loneliness	51.29 (7.72)	51.75 (9.37)	49.01 (8.88)	0.231	---
Perceived Hostility	49.22 (6.55)	50.65 (8.64)	48.99 (5.94)	0.395	---
Perceived Rejection	48.62 (7.90)	49.79 (9.29)	47. 63 (7.38)	0.346	---
**Average Perceived Stress Scale**	47.69 (7.86)	48.27 (9.55)	46.57 (7.75)	0.563	---

Analysis of Variance (ANOVA) followed by Tukey's Honestly Significant Difference test were used for statistical comparisons.

Supplemental Table 5. Alcohol drinking characteristics in subthreshold and AUD samples

	**Subthreshold (N=229)**	**AUD (N=262)**	***p***
% Women	47.60%	36.64%	0.018
Frequency of any alcohol use in the past 12 months[a]	4.10	3.34	<0.001
Frequency of drinking 5+ drinks in the past 12 months[b]	3.51	2.90	<0.001
Frequency drunk in the past 12 months[c]	2.97	2.45	<0.001
Max drinks consumed in a single day in the past 12 months[d]	3.26	4.11	<0.001
Drinks per drinking day in the past 12 months	2.58	3.13	<0.001

Welch's t-test and 2-sample test for equality of proportions with Yates continuity correction used for statistical comparisons. Subthreshold is defined as 1 criteria of alcohol abuse/dependence met. AUD is defined as 2+ criteria met. AUD = Alcohol Use Disorder. The Human Connectome Project assessed subjects for symptoms of alcohol abuse and dependence using the Semi-Structured Assessment for the Genetics of Alcoholism. Note that lower numbers on frequency variables indicate higher use levels: [a]4-7 days/week (1 if male, 2 if female), 3 days/week = 2, 2 days/week = 3, 1 day/week = 4, 1-3 days month = 5, 1-11 days/year = 6, never in past 12 months = 7; [b]3+ days/week = 1 if male, 2 if female; 1-2 days/week = 2; 1-3 days/month = 3; 1-11 days/year = 4; never = 5; [c]Frequency drunk in past 12 months: 1-7 days/week = 1 if male, 2 if female, 1-3 days/month = 2, 1-11 days/year = 3, never = ; [d]Max drinks consumed in a single day in the past 12 months: 1-2 = 1; 3-4 = 2; 5-6 = 3; 7-8 = 4; 9-10 = 5; 11-12 = 6 if male, 5 if female; 13+ = 7, 5 if female.

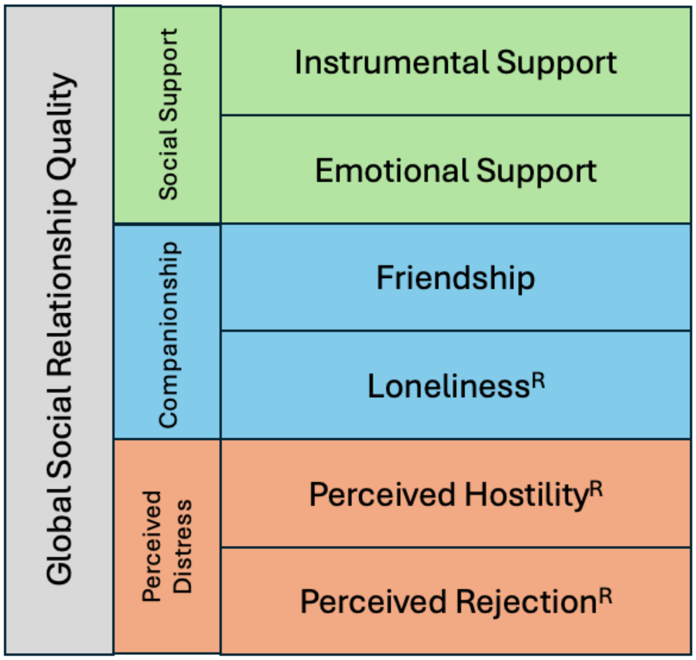

Supplemental Figure 1: Structure of NIH Social Relationship Quality (SRQ) scale. The SRQ scale is composed of three subdomains, each with two subscales. In the current study, we averaged across the six subscales to generate a global social relationship quality metric and between each subscale within a subdomain to generate a metric of companionship, social support, and perceived stress. R = reverse-scored in the present analysis.

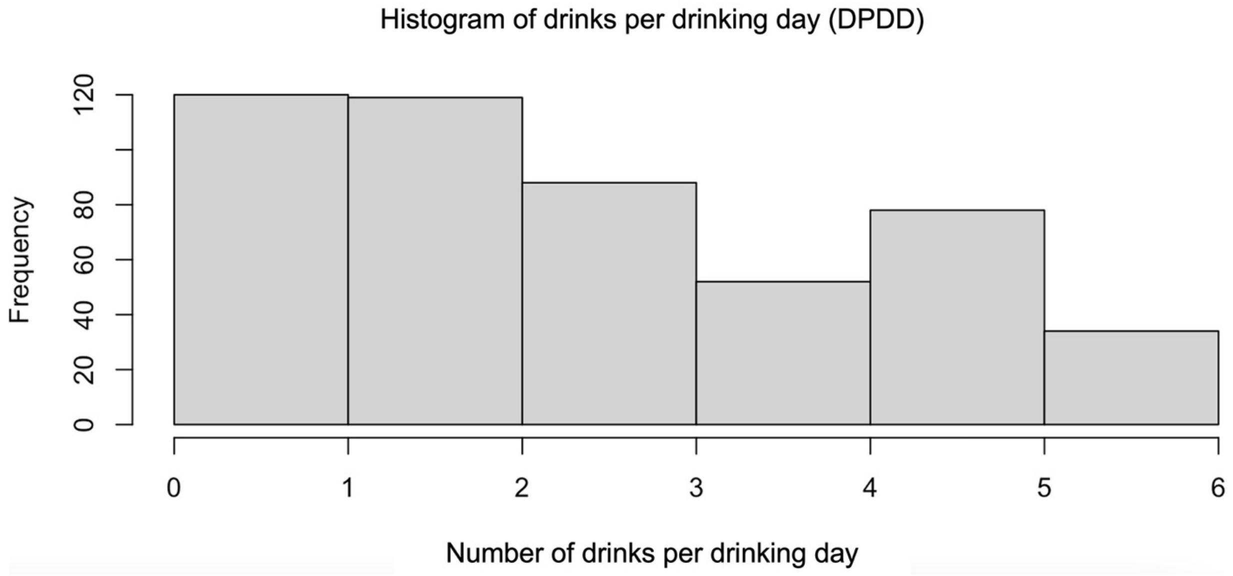

Supplemental Figure 2: Distribution of the drinks per drinking day (DPDD) in the full sample (N=491).

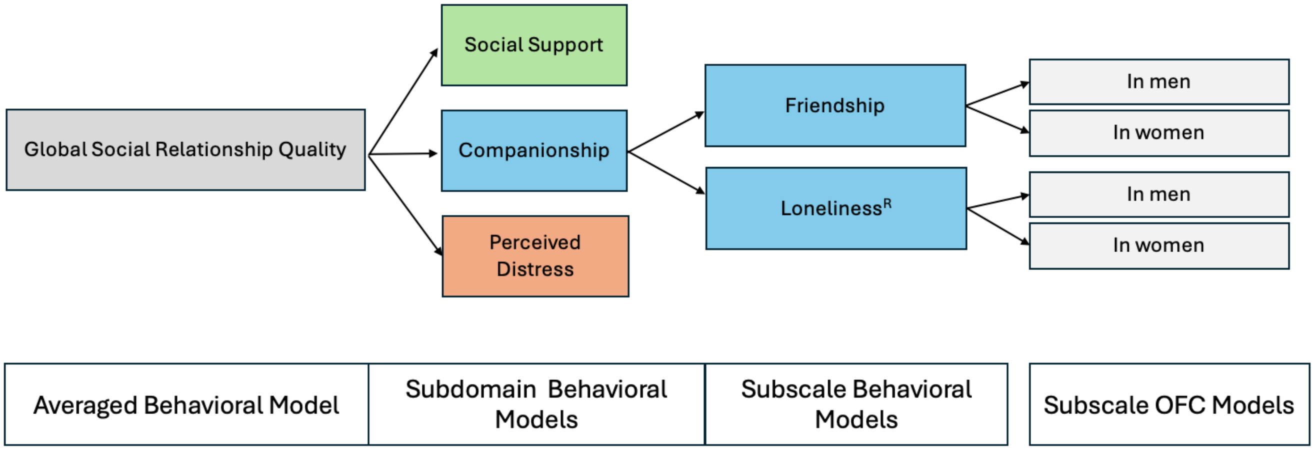

Supplemental Figure 3: Schema of data analytic plan of focal predictors.
First, we tested a moderated moderation model with global social relationship quality (SRQ) as the focal predictor. Second, we tested three models with each of the subdomains as the focal predictor, controlling for the other two subdomains. Third, given that only the model with 'companionship' as the focal predictor was significant, we tested two models with either friendship or loneliness as the focal predictor (controlling for the five other subscales). Finally, we tested in men and women separately models with either friendship or loneliness as the focal predictors (controlling for the other) and orbitofrontal cortex (OFC) reactivity as a moderator. All models covaried for race, age, ethnicity, and income, and the OFC model also controlled for posterior OFC reactivity. Interpretation of results accounted for multiple comparisons. OFC = orbitofrontal cortex; R = reverse-scored.

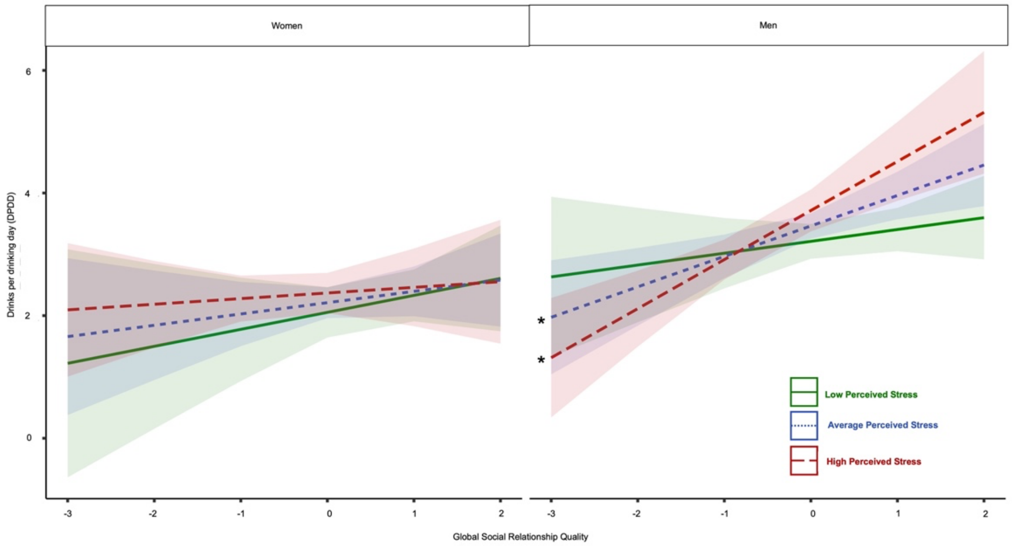

Supplemental Figure 4. Predicted values of drinks per drinking day by global social relationship quality, perceived stress, and sex/gender. Predictor and outcome variables were scaled for analysis; raw outcome values are shown here for interpretability. Shaded regions represent confidence intervals. * = a statistically significant ($p<0.05$) conditional effect.

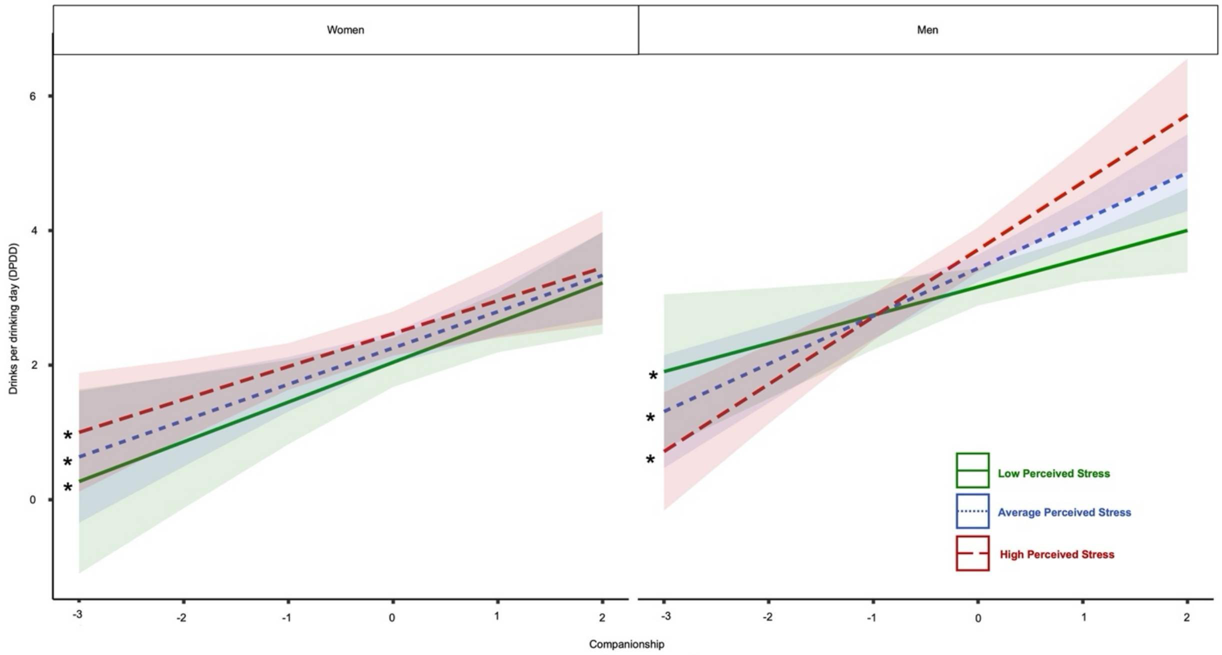

Supplemental Figure 5. Predicted values of drinks per drinking day by companionship, perceived stress, and sex/gender. Predictor and outcome variables were scaled for analysis; raw outcome values are shown here for interpretability. Shaded regions represent confidence intervals. * = a statistically significant ($p<0.05$) conditional effect.

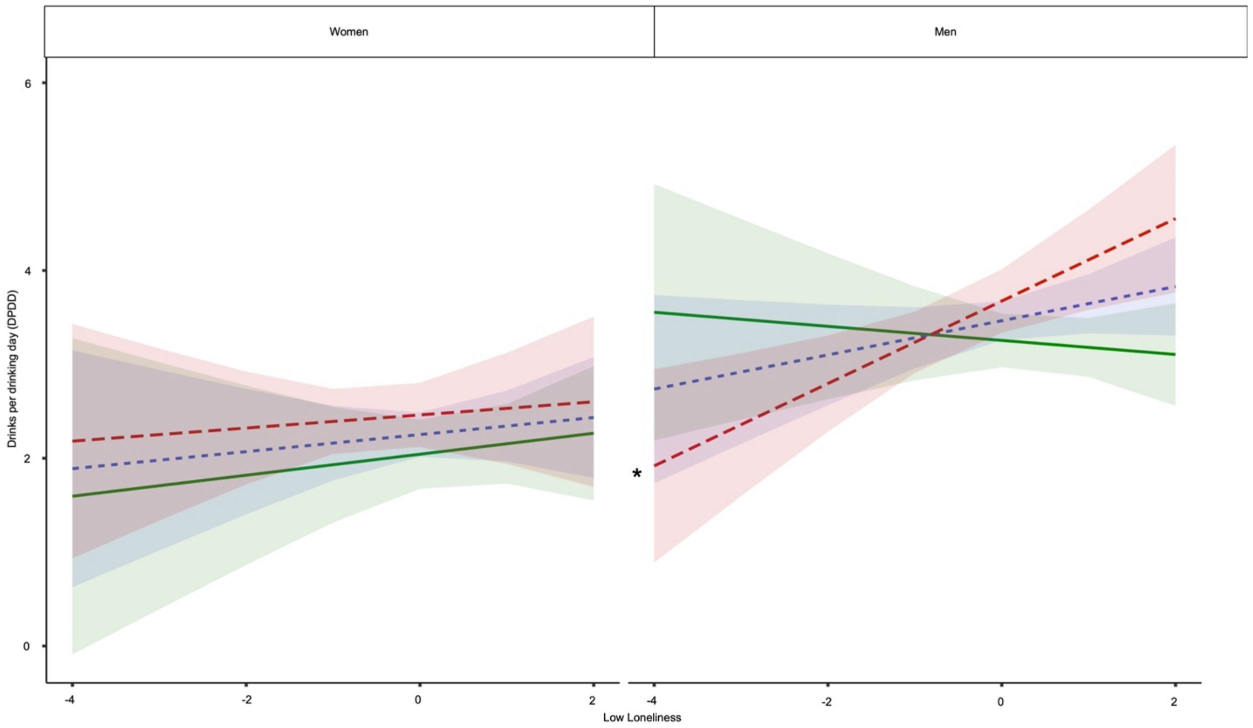

Supplemental Figure 6. Predicted values of drinks per drinking day by loneliness, perceived stress, and sex/gender. Predictor and outcome variables were scaled for analysis; raw outcome values are shown here for interpretability. Shaded regions represent confidence intervals. * = a statistically significant ($p<0.05$) conditional effect.

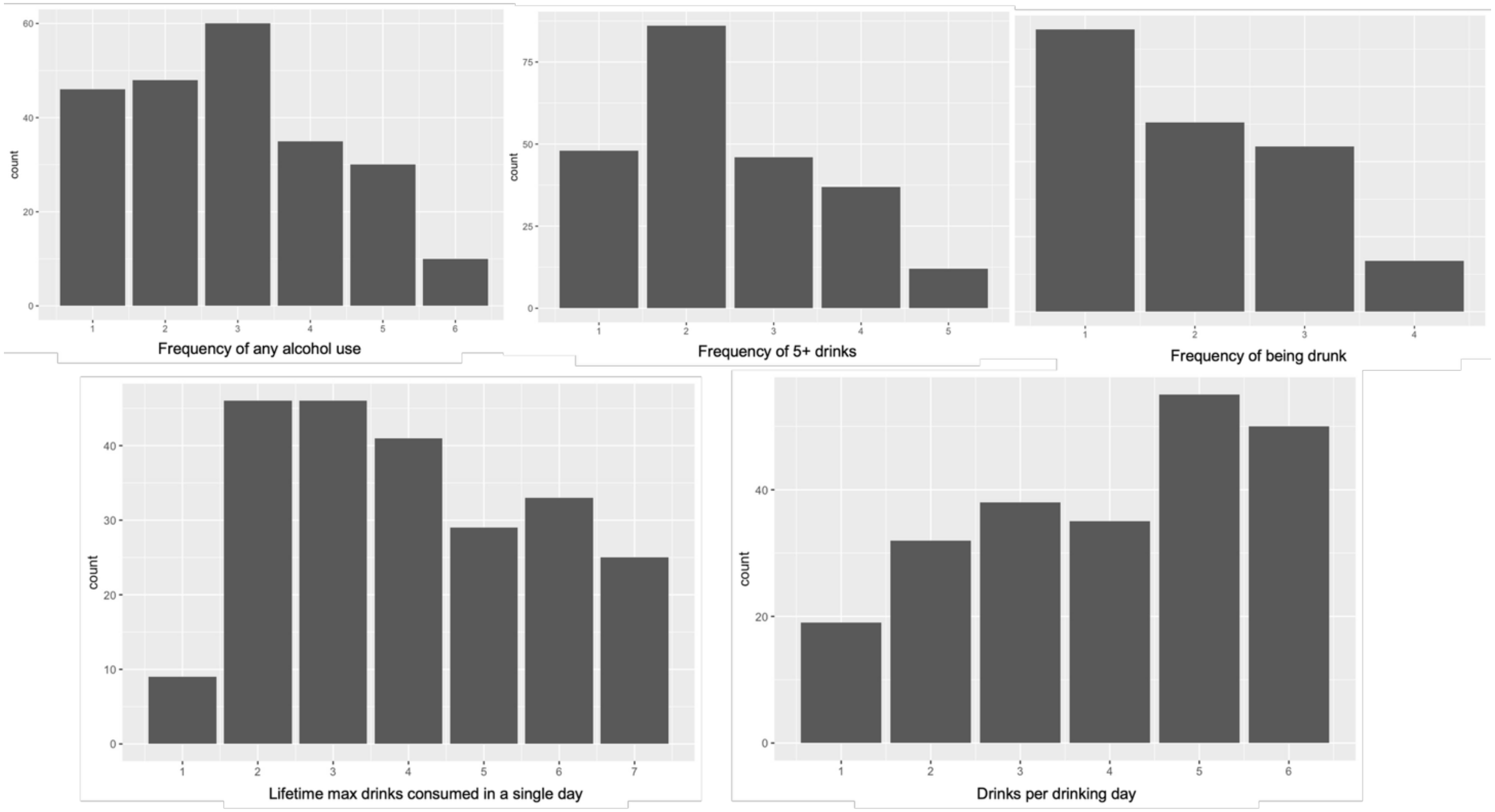

Supplemental Figure 7. Frequency distributions of alcohol use behaviors during the heaviest 12-month use period of the subthreshold group (frequency variables are reverse-coded). A frequency distribution of the subthreshold group's alcohol use behaviors during the heaviest 12-month period of use also demonstrated lower alcohol use levels for the subthreshold compared to the AUD group, see Supplemental Figure 8. Subthreshold is defined as 1 criterion of alcohol abuse/dependence met. AUD is defined as 2+ criteria met. AUD = Alcohol Use Disorder. The Human Connectome Project assessed subjects for symptoms of alcohol abuse and dependence using the Semi-Structured Assessment for the Genetics of Alcoholism. Note that lower numbers on frequency variables indicate higher use levels: Frequency of alcohol use: 4-7 days/week =1 if male, 2 if female, 3 days/week = 2, 2 days/week = 3, 1 day/week = 4, 1-3 days month = 5, 1-11 days/year = 6, never in past 12 months = 7; Frequency of 5+ drinks: 3+ days/week = 1 if male, 2 if female; 1-2 days/week = 2; 1-3 days/month = 3; 1-11 days/year = 4; never = 5; Frequency drunk in past 12 months: 1-7 days/week = 1 if male, 2 if female, 1-3 days/month = 2, 1-11 days/year = 3, never = 4 ; Max drinks consumed in a single day in the past 12 months: 1-2 = 1; 3-4 = 2; 5-6 = 3; 7-8 = 4; 9-10 = 5; 11-12 = 6 if male, 5 if female; 13+ = 7 if male, 5 if female.

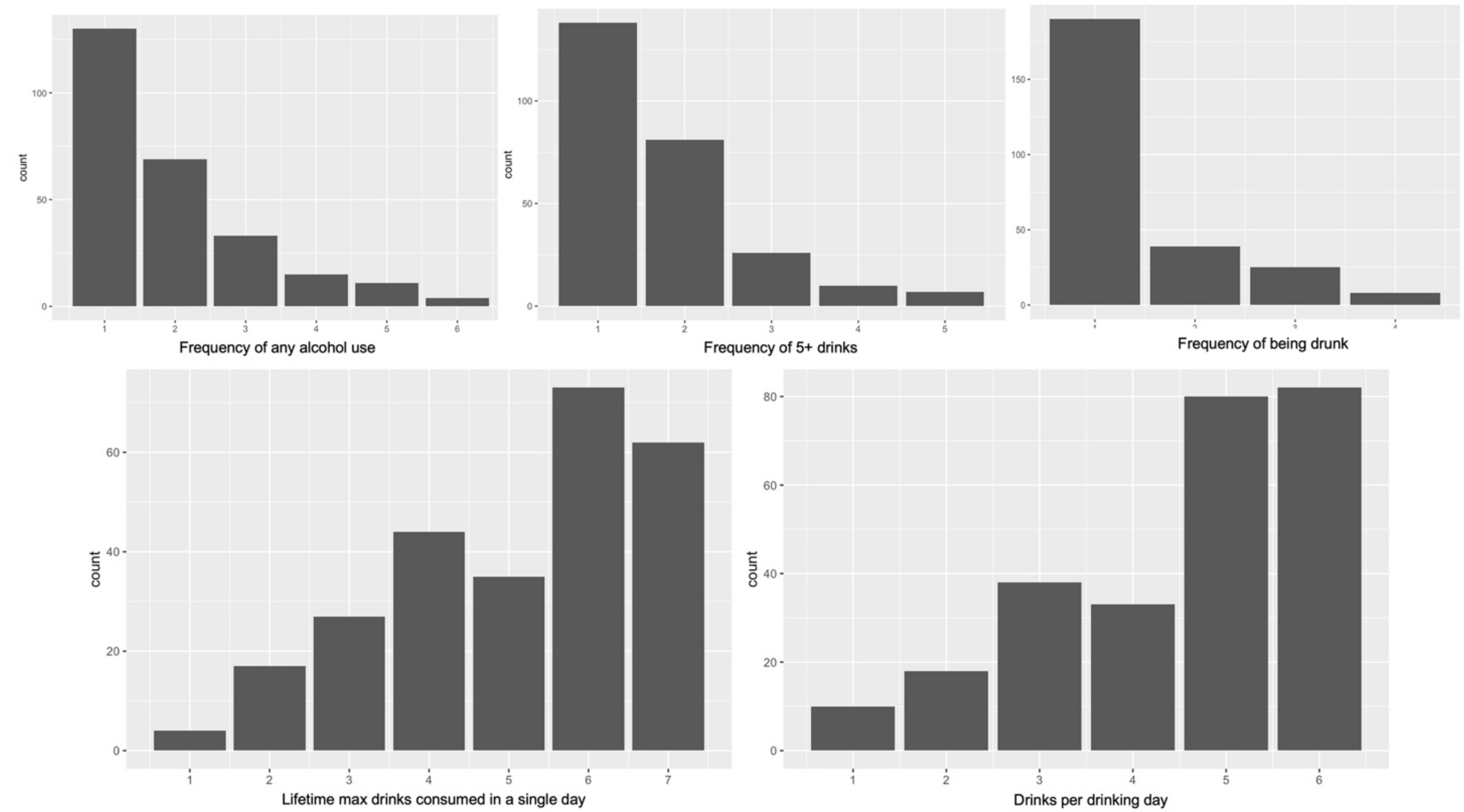

Supplemental Figure 8. Frequency distributions of alcohol use behaviors during the heaviest 12-month use period of the AUD group (frequency variables are reverse-coded). AUD is defined as 2+ criteria met. AUD = Alcohol Use Disorder. The Human Connectome Project assessed subjects for symptoms of alcohol abuse and dependence using the Semi-Structured Assessment for the Genetics of Alcoholism. Note that lower numbers on frequency variables indicate higher use levels: Frequency of alcohol use: 4-7 days/week =1 if male, 2 if female, 3 days/week = 2, 2 days/week = 3, 1 day/week = 4, 1-3 days month = 5, 1-11 days/year = 6, never in past 12 months = 7; Frequency of 5+ drinks: 3+ days/week = 1 if male, 2 if female; 1-2 days/week = 2; 1-3 days/month = 3; 1-11 days/year = 4; never = 5; Frequency drunk in past 12 months: 1-7 days/week = 1 if male, 2 if female, 1-3 days/month = 2, 1-11 days/year = 3, never = 4 ; Max drinks consumed in a single day in the past 12 months: 1-2 = 1; 3-4 = 2; 5-6 = 3; 7-8 = 4; 9-10 = 5; 11-12 = 6 if male, 5 if female; 13+ = 7 if male, 5 if female.

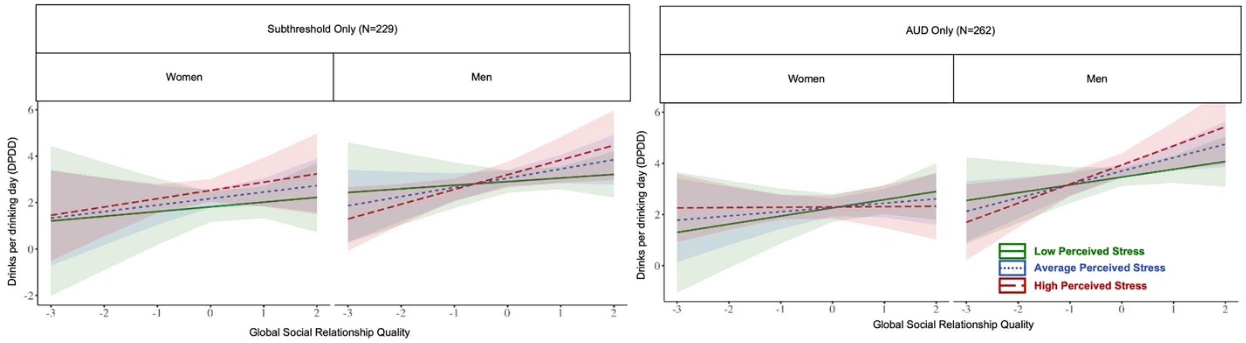

Supplemental Figure 9. **Predicted values of drinks per drinking day by global social relationship quality, perceived stress, and sex/gender tested separately in subthreshold and AUD groups.** The three-way interaction effect did not reach significance in either the subthreshold (*b* = -0.106, t(217) = -0.578, p=0.564) or AUD (*b* = -0.218, t(250) = -1.540, p=0.125) subsamples.

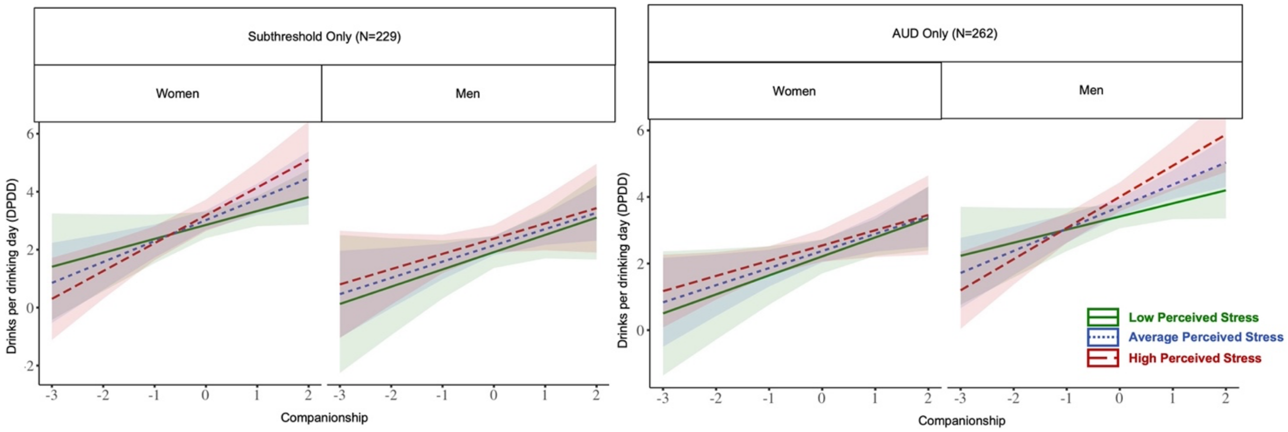

Supplemental Figure 10. **Predicted values of drinks per drinking day by companionship, perceived stress, and sex/gender tested separately in subthreshold and AUD groups.** The three-way interaction effect did not reach significance in the subthreshold (*b* = -0.179, t(215) = -0.876, p=0.382) sample but did trend toward significance in the AUD subsample (*b* = -0.191, t(248) = -1.906, p=0.058).

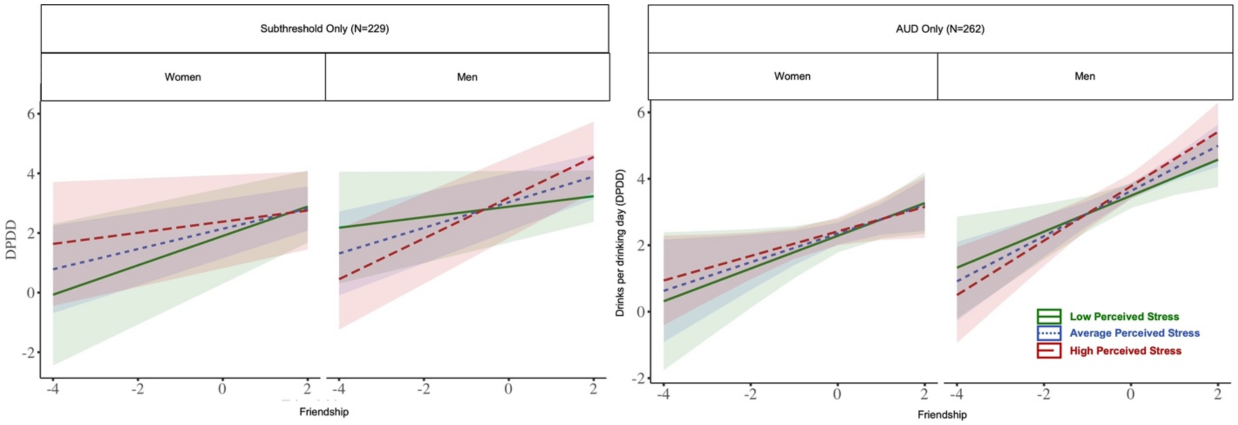

Supplemental Figure 11. **Predicted values of drinks per drinking day by friendship, perceived stress, and sex/gender tested separately in subthreshold and AUD groups.** The three-way interaction effect did not reach significance in either the subthreshold (*b* = -0.247, t(212) = -1.430, p=0.154) or AUD (*b* = -0.122, t(245) = -1.149, p=0.252) subsamples.

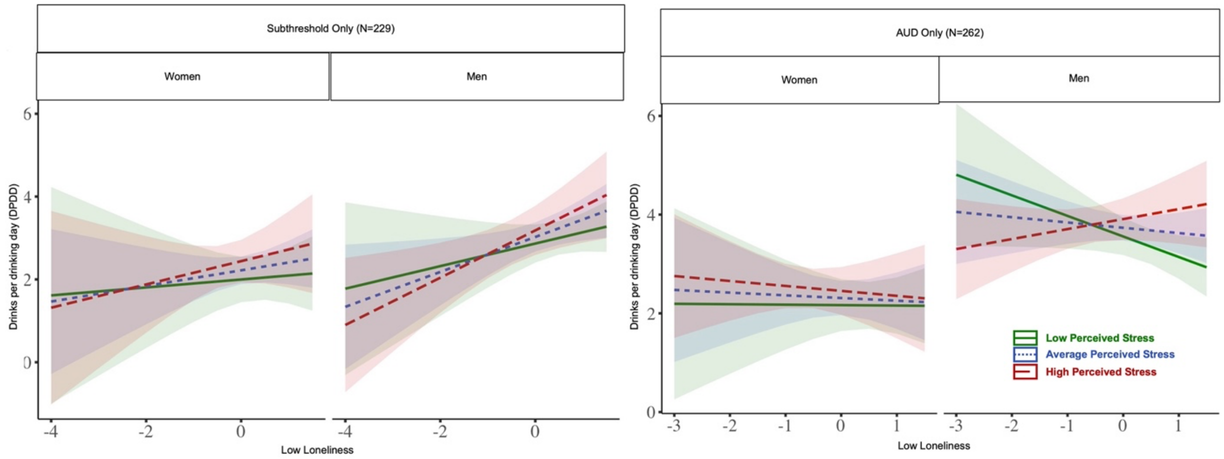

Supplemental Figure 12. **Predicted values of drinks per drinking day by loneliness, perceived stress, and sex/gender tested separately in subthreshold and AUD groups.** The three-way interaction effect did not reach significance in the subthreshold subsample (*b* = -0.035, t(212) = -0.23, p=0.816) but did trend toward significance in the AUD subsample (*b* = -0.216, t(245) = -2.345, p=0.0197).

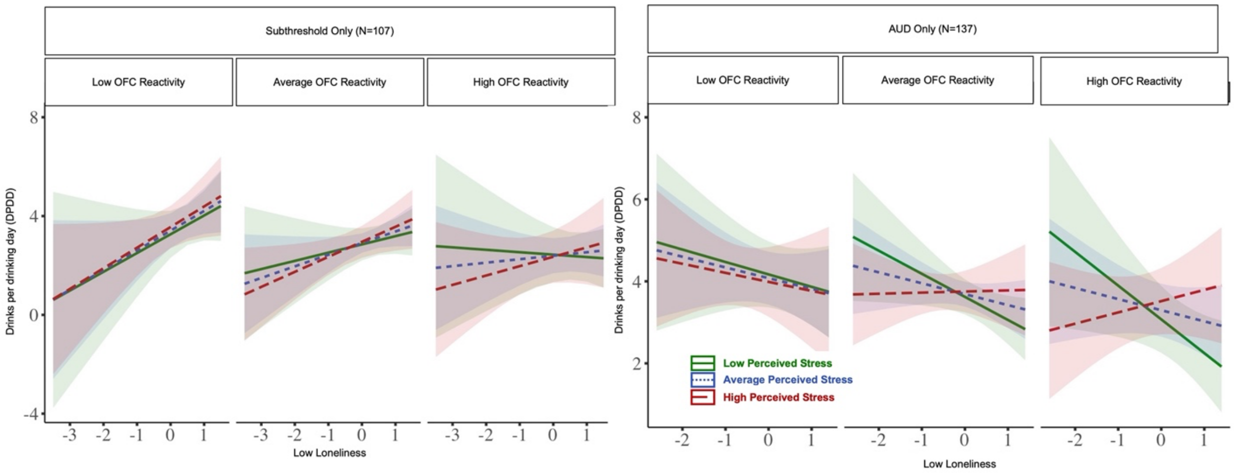

Supplemental Figure 13. Predicted values of drinks per drinking day by loneliness, perceived stress, and orbitofrontal cortex reactivity to emotional faces in men tested separately in subthreshold and AUD groups. The three-way interaction effect did not reach significance in the subthreshold subsample (*b* =0.067, t(89) = 0.477, p=0.635) but did trend toward significance in the AUD subsample (*b* = 0.146 t(119) = 1.774, p=0.079). OFC = orbitofrontal cortex.

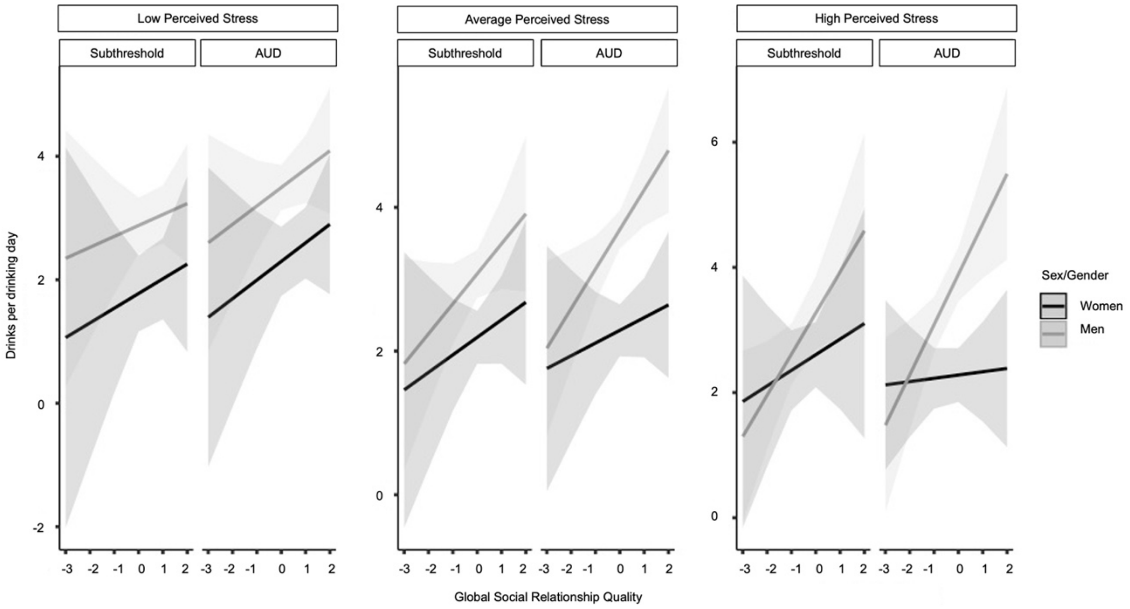

Supplemental Figure 14. Predicted values of drinks per drinking day by global social relationship quality, perceived stress, sex/gender, and threshold level. Predictor and outcome variables were scaled for analysis; raw outcome values are shown here for interpretability. Shaded regions represent confidence intervals.

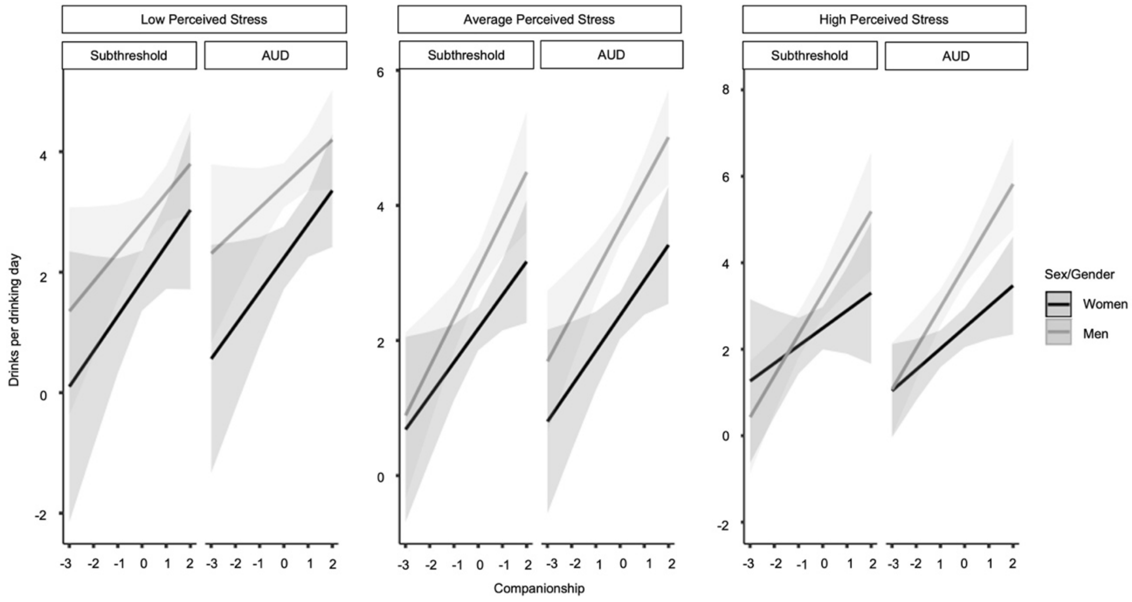

Supplemental Figure 15. Predicted values of drinks per drinking day by companionship, perceived stress, sex/gender, and threshold level. Predictor and outcome variables were scaled for analysis; raw outcome values are shown here for interpretability. Shaded regions represent confidence intervals.

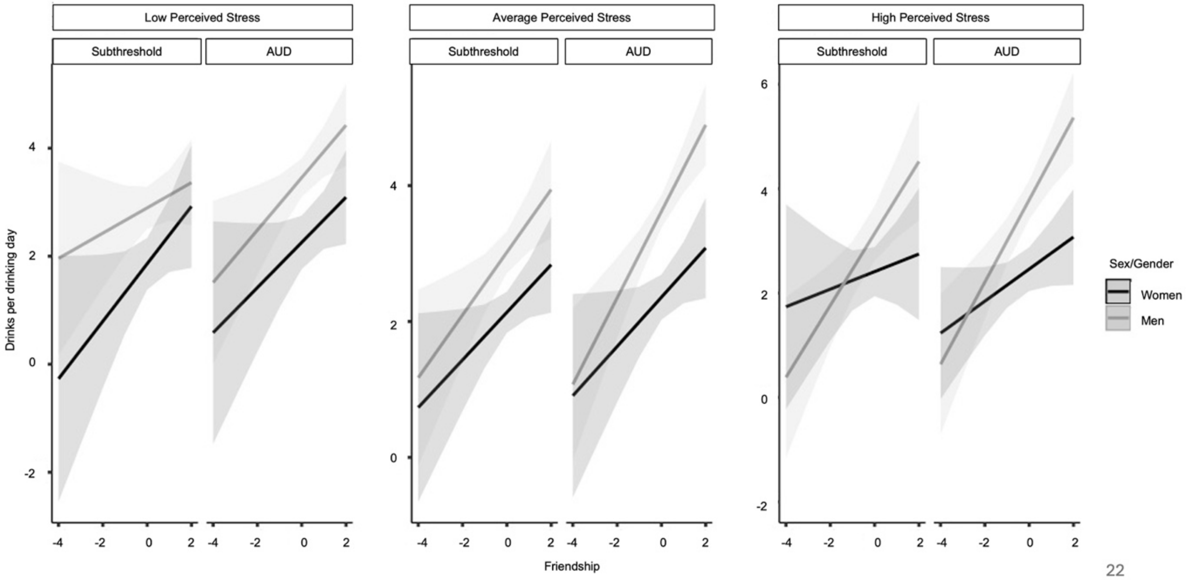

Supplemental Figure 16. Predicted values of drinks per drinking day by friendship, perceived stress, sex/gender, and threshold level. Predictor and outcome variables were scaled for analysis; raw outcome values are shown here for interpretability. Shaded regions represent confidence intervals.

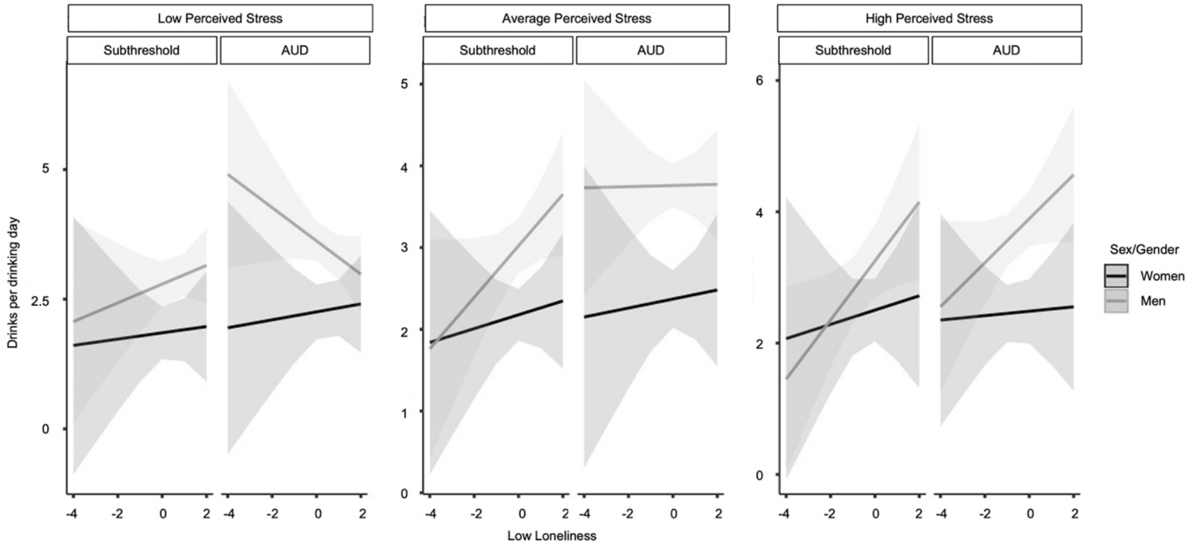

Supplemental Figure 17. Predicted values of drinks per drinking day by loneliness, perceived stress, sex/gender, and threshold level. Predictor and outcome variables were scaled for analysis; raw outcome values are shown here for interpretability. Shaded regions represent confidence intervals.

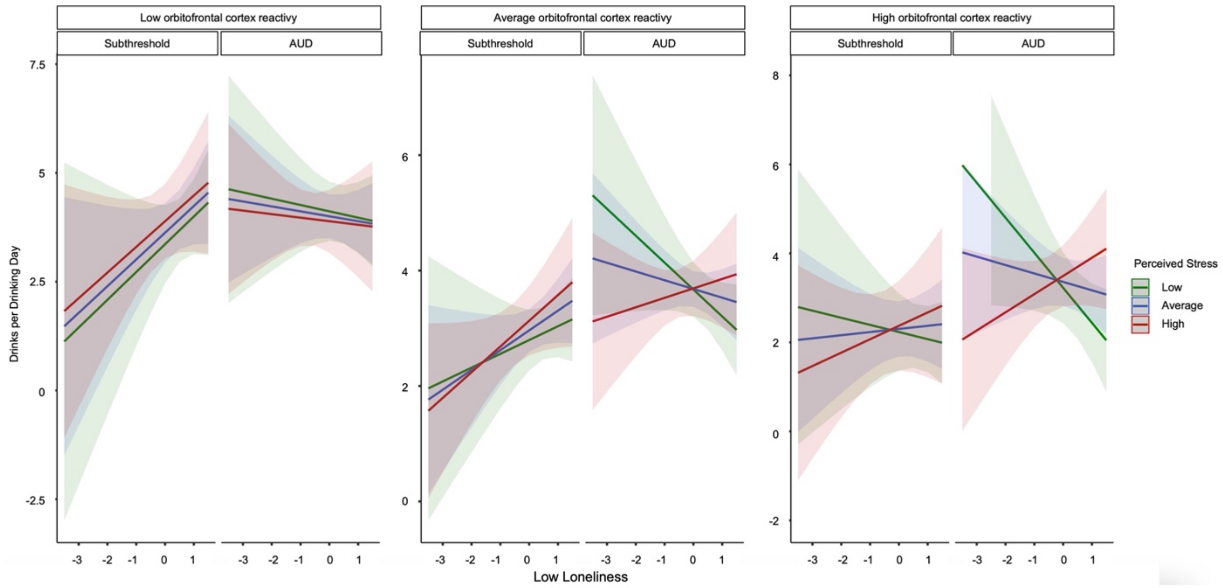

Supplemental Figure 18. Predicted values of drinks per drinking day in men by loneliness, perceived stress, orbitofrontal cortex reactivity, and threshold level. Predictor and outcome variables were scaled for analysis; raw outcome values are shown here for interpretability. Shaded regions represent confidence intervals.

Supplemental Citations

Cao, Jing, and Song Zhang. "Multiple comparison procedures." *Jama* 312.5 (2014): 543-544

Staffa, Steven J., and David Zurakowski. "Strategies in adjusting for multiple comparisons: a primer for pediatric surgeons." *Journal of Pediatric Surgery* 55.9 (2020): 1699-1705
